# Network analysis of atherosclerotic genes elucidates druggable targets

**DOI:** 10.1186/s12920-022-01195-y

**Published:** 2022-03-03

**Authors:** Sheuli Kangsa Banik, Somorita Baishya, Anupam Das Talukdar, Manabendra Dutta Choudhury

**Affiliations:** Department of Life Science and Bioinformatics, Assam University, Silchar, India

**Keywords:** Atherosclerosis, Network medicine, Gene network analysis, Cytoscape, STRING, AGT, LPL, ITGB2, IRS1, miRNA, MIENTURNET

## Abstract

**Background:**

Atherosclerosis is one of the major causes of cardiovascular disease. It is characterized by the accumulation of atherosclerotic plaque in arteries under the influence of inflammatory responses, proliferation of smooth muscle cell, accumulation of modified low density lipoprotein. The pathophysiology of atherosclerosis involves the interplay of a number of genes and metabolic pathways. In traditional translation method, only a limited number of genes and pathways can be studied at once. However, the new paradigm of network medicine can be explored to study the interaction of a large array of genes and their functional partners and their connections with the concerned disease pathogenesis. Thus, in our study we employed a branch of network medicine, gene network analysis as a tool to identify the most crucial genes and the miRNAs that regulate these genes at the post transcriptional level responsible for pathogenesis of atherosclerosis.

**Result:**

From NCBI database 988 atherosclerotic genes were retrieved. The protein–protein interaction using STRING database resulted in 22,693 PPI interactions among 872 nodes (genes) at different confidence score. The cluster analysis of the 872 genes using MCODE, a plug-in of Cytoscape software revealed a total of 18 clusters, the topological parameter and gene ontology analysis facilitated in the selection of four influential genes viz., AGT, LPL, ITGB2, IRS1 from cluster 3. Further, the miRNAs (miR-26, miR-27, and miR-29 families) targeting these genes were obtained by employing MIENTURNET webtool.

**Conclusion:**

Gene network analysis assisted in filtering out the 4 probable influential genes and 3 miRNA families in the pathogenesis of atherosclerosis. These genes, miRNAs can be targeted to restrict the occurrence of atherosclerosis. Given the importance of atherosclerosis, any approach in the understanding the genes involved in its pathogenesis can substantially enhance the health care system.

**Supplementary Information:**

The online version contains supplementary material available at 10.1186/s12920-022-01195-y.

## Background

Atherosclerosis is a multifactorial inflammatory disease of the arteries. It is the major cause of most cardiovascular diseases like myocardial infarction, peripheral artery disease and stroke [86]. According to World Heart Federation, cardiovascular disease is the leading cause of mortality worldwide, causing 17.9 million deaths per year (www.world-heart-federation.org/world-heart-day/world-heart-day-2019/cvds, accessed on 11/02/2021).Atherosclerosis is characterized by the accumulation of modified low density lipoprotein (LDL) in the arteries that are exposed to turbulent flow of blood, thereafter triggers a series of inflammatory responses. Initially monocytes are recruited to bind to the endothelium and then they migrate into the sub endothelial cells and convert to macrophages that engulf the modified LDL and form foam cells. This induces proliferation of smooth muscle cell, accumulation of extra cellular matrix protein and results in the formation of atherosclerotic plaque that hinders the regular flow of blood [13, 27, 67].

Studies have shown that the pathophysiology of atherosclerosis involves the interplay of a number of genes and metabolic pathways, hence they are greatly targeted to overcome the atherosclerotic complications [16]. The cholesterol transport system is an important component in the regulation of atherosclerosis and the genes associated with itlike APOA1, ABCA1, ABCG1, LCAT, APOB, CETP, LPL, PPARγ, LXR, SR-A, FoxO1, PCSK9,etc. were investigated immensely to gain scientific insights in order to establish them as a target [48, 86]. Also, the genes that are involved with the inflammatory responses like VCAM1, MCP1, M-CSF, IL-1β, TNF α and β, IL-6, M-CSF, MCP-1, IL-18 and CD-40L, etc. were thoroughly studied to elucidate their role in atherosclerosis[21]. Thus, from an array of genes, prioritization of gene/s that is/are key regulator of atherosclerosis becomes difficult from the translational aspect.

In this regard, Network Medicine (NM), a very recent and promising discipline that aids in the identification of disease genes, pathways and the probable targets for drugs can be utilized. This NM approach takes under consideration the system biology and network science to provide a deeper insight in the perturbation of a disease in a holistic way. NM encompasses the integration of large biological data sets (genomics, proteomics, etc.) and creates desired network type (protein–protein interaction, metabolomics, etc.) to elucidate the connections among the biological components and thereafter revealing the influential pathophenotype [12, 20, 30, 58]. Here under the framework of NM, gene network analysis was conducted to predict the most influential gene/s in the pathogenesis of atherosclerosis. This *in-silico* approach facilitates to reconstruct the interactions between genes, proteins, signaling pathways associated with metabolic disorders by using low and high throughput experimental and computational data having statistical significance [72]. In this study, a protein–protein interaction network of the concern disease involving gene/protein and their associated partners was constructed. Further based on the cluster formation and gene ontology analysis, the key regulatory gene can be identified [3, 10]. Gene network analysis has been successfully used by Sarajlic et al. to identify the ‘driver genes’ that are involved in cardiovascular diseases [65]. In coronary artery disease the Combined Network Topological Features was explored to identify the crucial gene involved with the disease [88]. Gene network analysis has also employed in predicting novel genes/therapeutic drug targets of human genes infected by COVID 19, meniere’s disease, chronic obstructive pulmonary disease It has also helped in the identification of driver epigenetic factor, tumor suppressor genes and the genes responsible for glioblastomaTherefore, gene network analysis facilitates to design drug for various infections and also for varioius metabolic diseases [28, 55, 90, 92 40, 19, 39]. It also aids in identification of sub-networks of diseased genes that are associated with varied disorders by interactome based network analysis [56]. An investigation was conducted on the switch genes of different cancer types caused by BRAF^V600E^ mutant in order to understand the mechanisms of the varied response exhibited by a drug, vemurafenib [26]. Thus, it can be seen that gene network analysis has a wide range of implication in the biological domain.

Gene network analysis assists in the identification of influencial genes and these genes are inturn regulated by miRNA. Nowadays, clinical researches exploit miRNA as a therapeutic target to treat various diseases like coronary heart disease, hepatitis C, liver cancer, diabetes, etc. miRNAs are the short non-coding RNA molecules formed from a long primary RNA transcript and are expressed in the peripheral blood, cerebrospinal fluid, saliva, urine, and other biological samples. A single miRNA has the potential to target a large number of mRNA and regulate the expression of gene. The treatment regime includes either blocking the expression of a specific miRNA or compensating the expression loss of miRNA [31,[76]. Various studies have shown that when miRNA was targeted the pathophysiology of CVD could be obsoleted, like when the expression of miR-378 was enhanced cardiac congestion could be prevented and when the expression of miR-133 was inhibited the occurrence of hyperthrophy was reduced [18], the downregulation of miR-1 and miR-26 and the upregulation of miR-21 and miR-328 along with the alteration of other miRNAs have helped in the reduction of Atrial fibrillation [82].

## Method

### Retrieval of genes

The genes associated with atherosclerosis were retrieved from National Centre for Biotechnology Information (NCBI) database (https://www.ncbi.nlm.nih.gov/gene; Accessed on 22/12/2020) using the query words “atherosclerosis, *Homo sapiens*”.

### Protein–protein interaction

The protein–protein interaction (PPI) data were generated by using the freely available database STRING (Search Tool for the Retrieval of Interacting Genes/Proteins) version 11 (https://string-db.org/). STRING encompasses both physical and functional aspects of proteins and takes into account evidences from experimental works, databases mining, pathway databases, co-expressed and neighborhood gene analysis, fusion and co-occurrence of protein association/distribution analysis. The interactions among the proteins are depicted by confidence scores that range between 0 and 1. The highest confidence score lies between 0.9 and 1, high confidence score between 0.7 and 0.9, medium confidence score between 0.4 and 0.7 and low confidence score is less than 0.4. In the network, each gene is designated as node and the interactions between the nodes are designated as edge [4, 69, 70]

### Construction of gene interaction network

The visualization model of biological network is generated by using Cytoscape (3.4.0) Software. It is afreely available software with various other plug-in apps viz., MCODE for clustering analysis, BiNGO/ClueGo for functional enrichment analysis, AgilentLiteratureSearch for literature mining, etc. The network obtained from PPI analysis was imported to Cytoscape in a network specific format such as SIF (Simple Interaction File). Cytoscape merges, visualize and analyse the STRING networks using various algorithm for network layout like spring-embedded layout, hierarchical layout, and circular layout [64, 70].

### Gene clustering analysis

MCODE (Molecular COmplexDEtection), a Cytoscape plug-in is a density-based clustering algorithm that identifies the clusters of densely connected nodes from the network by generating a score and rank. It operates in three levels: Firstly it weighs all nodes using *k*-core value. Secondly, centering the highest weighted node the cluster is predicted. Thirdly, it undergoes post-processing depending on core value. The resulting complexes are then scored, ranked accordingly and graphical modules are obtained [9, 64], and hence enhances the reliability of MCODE. Clustering analysis by MCODE has aided in the identification of cruicial genes in the pathophysiology of atherosclerosis and also in other diseases [57, 73].

### Topological parameter analysis

The topological parameters analysis depicts the arrangement of interacting nodes within a network. Cytoscape plugin Network Analyzer takes into account topological parameters like node degree betweeness centrality, and average shortest pathlength. The number of edges extending out of a node determines its degree. Significant biological interactions are considered to be associated with high node degree. Average Shortest Pathlength is the measure of the least distance between two nodes. Betweenness centrality of a node is the summation of all the shortest pathlength from onenode to another connected either directed or indirectly [6, 84].

### Gene ontology and pathway interrelation analysis

ClueGo in association with CluePedia were used for gene ontology and pathway enrichment analysis. This cytoscape plug-in facilitates the prediction of biological pathway conferred by genes which can be visualized as networks of functionally grouped terms. It uses the precompiled GO terms to calculate the enrichment analysis for GO terms, GO term fusion, mid-*p*-values, Bonferroni step-down method and p value less than equals to 0.5 were used, keeping predefined kappa threshold value [15]

### miRNA target prediction

The prediction of miRNA that targets the obtained influential genes was facilitated by the web tool MIENTURNET (MicroRNA ENrichment TURned NETwork). This tool takes under consideration both the computational and experimental evidences from TargetScan and miRTarBase websites respectively to predict the interaction of miRNA on target gene. The input for MIENTURNET could be either gene list or miRNA list and a network is obtained that is statistically significant. The analysis of the network was done by computing the topological parameters, and performing functional enrichment analysis [41].

## Result

### Construction of gene networks of atherosclerotic genes

The query for “*Atherosclerosis, Homo sapiens*” in the NCBI database revealed a total of 988 genes that were involved in atherosclerosis (Additional file 1: Table S1). The input of all 988 genes in STRING revealed that 96 genes were not found in the database. Thus, networks with 892 genes were constructed at different confidence scores. At medium confidence score 22,693 protein–protein interactions were found among 872 nodes (Additional file 2: Table S2c) whereas at high confidence score 8314 protein–protein interactions were found among 777 nodes (Additional file 2: Table S2b), and at highest confidence score 5248 protein–protein interactions were obtained among 675 nodes (Additional file 2: Table S2a).

### Cluster analysis

Using MCODE, the densely associated nodes in the protein–protein interaction network were obtained as clusters. Total 18 clusters were obtained and were ranked based on their scores (density to node ratio). Out of 872 nodes, only 465 nodes having 6607 edges (interaction) were associated with cluster formation whereas others remained unclustered. The 18 clusters along with the scores and name of associated nodes are listed in Table 1. The individual clusters are depicted in Additional file 8: Figure S1.

**Table 1:** List of clusters as obtained by MCODE analysis

Cluster	Score (Density*#Nodes)	Nodes	Edges	Node IDs
1	69.631	158	5466	VEGFA, VEGFC, AKT1, PDGFRB, STAT3, NLRP3, NOS3, TLR7, CASP3, STAT1, CD28, CD86, JUN, CD40LG, CD40, PTEN, APOB, ESR1, RHOA, APOE, APOA1, HIF1A, TP53, MAPK1, KDR, MAPK3, PTGS2, ACE, AGTR2, FASLG, MMP2, ACKR3, C5, FLT1, BDKRB1, C3, GNB3, GPR55, ANXA5, MTNR1B, GPER1, P2RY12, SUCNR1, IL6R, GRM8, SAA1, IFNG, HCAR2, FPR1, FPR2, CNR1, CASR, APLN, ANXA1, NGF, CCL28, APLNR, CCR2, NPY, CXCR5, CCR5, CXCR6, PPBP, PECAM1, CXCR4, PF4, TLR4, CXCL5, TLR6, CXCL16, CCL5, CXCL1, CXCL12, CXCL8, TNFRSF1A, TNFSF11, MPO, IL18, ELANE, NOTCH1, IL15, ADAM10, LGALS1, SERPIND1, TIMP1, PROC, IGFBP5, FGF23, APOA2, ICAM1, STC2, KITLG, F5, MIA3, VCAM1, QSOX1, APOA5, APOL1, TNC, MMP9, PCSK9, AHSG, SERPINA1, CP, CD44, MMP3, C4A, SPP1, TLR2, IGFBP1, CSF1, TLR1, MFGE8, IGFBP3, CST3, GAS6, PLAU, MMP1, ALB, FN1, IL6, CDH1, ITGB1, BDNF, TNF, HMOX1, IL17A, VWF, CCL2, ADIPOQ, IL10, IL1B, CRP, IDO1, LEP, PPARG, CD68, FOXP3, SELL, INS, CD34, PTPRC, PROM1, ENG, CCL11, TNFSF13B, EDN1, HMGB1, SELE, RELA, ELN, SRC, JAK2, HGF, TGFB1, IGF1, SERPINE1, PLG
2	12.615	66	410	KLRK1, BMP2, IGF2, IL9, SOCS3, SIRT1, HBEGF, CAT, JAG1, SELP, SNAI1, GPR29, LCN2, TBX21, MIF, NOS2, MMP10, MMP7, MTOR, CYBB, PLAUR, MMP14, AR, CTNNB1, EGF, IL7R, AGTR1, SOD2, TNFSF10, ADAM17, GPT, CCL17, F3, FCGR2A, IL5, SYK, CDC42, RETN, CCL22, CX3CL1, CD163, ANGPT1, CD69, ANGPT2, PGF, KIT, F2, NFKB1, CXCR2, CX3CR1, CXCR3, SOCS1, HAVCR2, IGF1R, IL33, CDH5, CXCL13, CDKN2A, HSPA4, BGLAP, LGALS3, NOD2, GJA1, IL1A, CLEC7A, IL1RN
3	10.217	84	424	CTSB, IL22, IL23R, IL23A, BRCA1, OSM, MMP8, CDKN1B, SOD1, TREM1, HSPA5, KLF4, CAMP, IRS1, TERT, NOX1, NOX4, NCF1, JAK1, DCN, ITGB2, IL2RA, CD4, FOXO1, WNT5A, VLDLR, ILK, AVP, FOXO3, IKBKB, LYN, SELPLG, PLTP, ITGB3, ABCB1, TIMP3, LCAT, B2M, PLAT, TXN, ALOX5, TNFRSF11B, TNFRSF9, NR3C1, AGT, ITGA4, AHR, LPL, GGT2, GCG, VDR, P2RY2, APOA4, SAA2, VIMP, GGT1, CETP, AGER, APOC3, PON1, UTS2, HPR, MSR1, KISS1R, APOC1, GHSR, EDNRA, APOC2, UTS2R, PLA2G7, UTS2B, EDN3, MCAM, KISS1, APOBR, CYSLTR1, EDNRB, LTB4R, LIPC, NFE2L2, APOH, TNFAIP3, COL18A1, TNFSF4
4	5.3	41	106	LEPR, DICER1, CDKN3, PGLYRP1, BMP4, HSPD1, HSPB1, ITGA2B, PTX3, DKK1, ADIPOR1, ITGA2, UCP1, PTH, CHIT1, LIPG, CALCA, HPSE, NEU1, EZH2, CYP19A1, BGN, CD14, SREBF2, NPC1L1, MTTP, DNMT1, EPHA2, SIRT6, ITGAV, CTSK, VIM, SCARB1, ACP5, PPARA, ESR2, STK11, VCL, ABCG8, ABCG5, SREBF1
5	5.282	40	103	CTSL, SCD, GC, NAMPT, UCP2, LTA4H, FADS1, RBP4, FABP4, KLF2, MDK, FGF21, TNFAIP6, ANGPTL3, ADIPOR2, ADM, ANGPTL4, CTSS, HLA-DRB1, MAPK7, HSPG2, GATA2, GHRL, HP, LPA, OLR1, PLCG1, ADRB2, F2R, LDLR, ABCA1, RARRES2, HMGCR, IRS2, NR1H3, ABCG1, ACTA2, NOTCH2, S100A9, NRG1
6	4.8	6	12	LTC4S, TBXAS1, ALOX15, ALOX12, PLA2G6, PLA2G2A
7	3.333	4	5	HABP2, F12, HGFAC, PROZ
8	3.333	4	5	DHX38, THOC5, HNRNPC, DHX15
9	3.2	6	8	ACSL1, FABP3, CPT1A, NR4A1, UBE2I, NCOA2
10	3	3	3	MSTN, MEF2C, PPARGC1A
11	3	7	9	NUMB, CLTCL1, AAK1, PSMA6, PSMD6, SCARB2, LTBR
12	3	3	3	ADAMTS3, ADAMTS1, COMP
13	3	3	3	NAT2, GSTM1, GSTO1
14	3	3	3	DDAH2, DDAH1, ARG2
15	3	3	3	PAFAH1B2, PLA2G10, PAFAH1B3
16	3	3	3	RNF111, RNF213, HERC6
17	2.846	27	37	ADAMTS4, ADAMTS5, TLN1, ADAMTS13, CAP1, GSTP1, MAN2B1, THBS2, AKR1B1, RIPK2, CHI3L1, PIK3CB, ORMDL3, CD36, CDK5, PLIN2, XDH, ELAVL1, CTSD, YWHAZ, ADAM8, PRDX1, TNFSF12, ARSB, TRAF2, TNFRSF25, CTSC
18	2.667	4	4	GLS2, TSHB, GCLC, TXNRD2

### Network analysis

The Cytoscape tool, network analyzer facilitated in the analysis of all the 18 clusters. The topological parameters like node degree distribution, betweenness centrality, and average shortest pathlength were analysed for all the clusters. The topological parameters of all the clustered nodes are listed in Additional file 3: Table S3.

### Biological function and pathway analysis

**Fig. 1:**
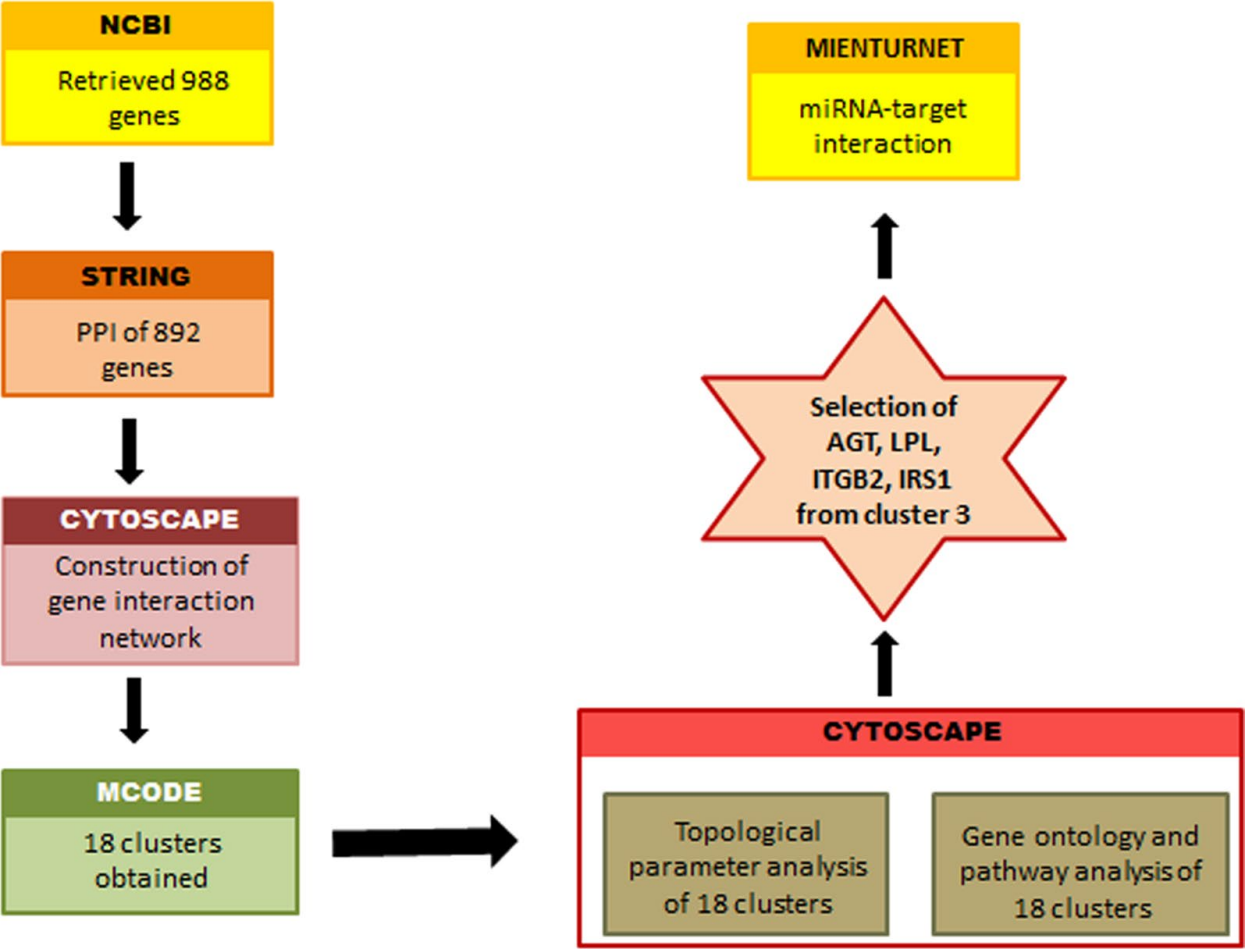
A flowchart of the network analysis

ClueGo in association with CluePedia, a cytoscape plug-in was used for functional enrichment analysis. Using the default network specificity of the plug-in, the enrichment of all the 18 clusters was done. Out of which Cluster 1, 2, 3, 4, 5, 6 were found to be functionally enriched. For cluster 1, 310 Gene Ontology (GO) terms were obtained; for cluster 2, 26 GO terms were obtained; for cluster 3, 27 GO terms were obtained; for cluster 4, 3 GO terms were obtained; for cluster 5, 8 GO terms were obtained, and for cluster 6, 1 GO term was obtained. The list of all the GO terms of 6 clusters alongside the biological processes and genes involved are given in Additional file 4: Table S4. A flow chart of the network analysis has been depicted in Fig. 1.

### MiRNA-target interaction

**Fig. 2:**
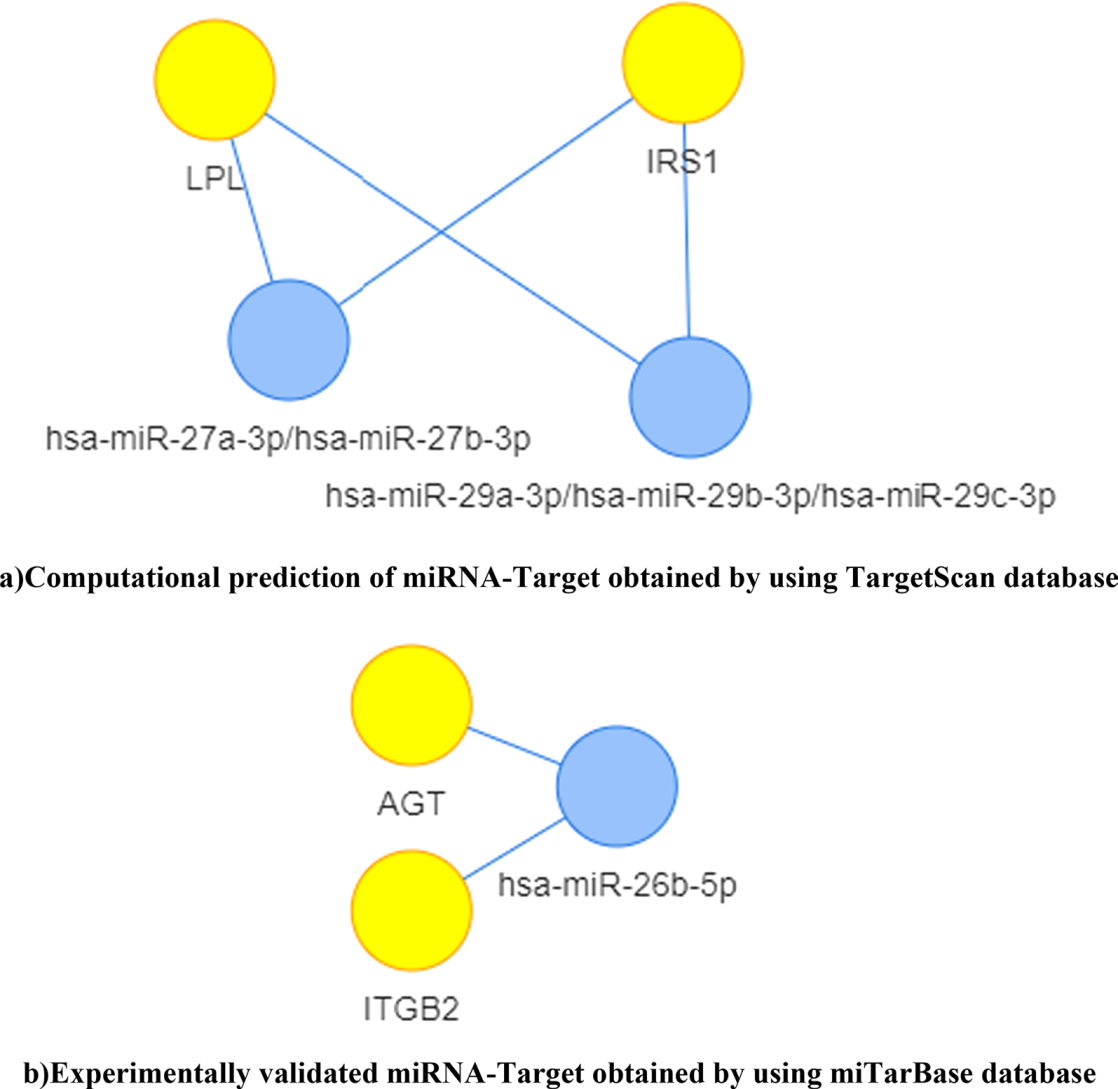
miRNA-Target interaction using MIENTURNET

The genes AGT, LPL, IRS1, and ITGB2 were uploaded as official gene symbol in MIENTURNET web tool. The statistical analysis was performed by miRNA-Target enrichment using both TargetScan and miRTarBase database to over-represent the miRNA-Target interaction. TargetScan generated miRNA family hsa-miR-29a-3p/hsa-miR-29b-3p/hsa-miR-29c-3p and hsa-miR-27a-3p/hsa-miR-27b-3p. These miRNAs interacted with IRS1 and LPL by binding with seeds (conserved regions) AGCACCA and UCACAGU respectively. Whereas, miRTarBase generated one miRNA, hsa-miR-26b-5p and this interacted with IGBT2 and AGT (Fig. 2, Table 2). The topological parameters of the nodes are provided in Additional file 5. The disease ontology of the miRNAs revealed a total of 124 and 95 GO terms using TargetScan and miTarBase database respectively (Additional file 6). Of which 30 GO terms obtained from TargetScan and 16 GO terms obtained from miTarBase database are associated with atherosclerosis either directly or indirectly.

**Table 2:** miRNA-Target interaction

Seed	miRNA family	*p* value	Odd ratio	Number of interactions	Target Gene 1	Target Gene 2
**Mienturnet Enrichment results using TargetScan**						
AGCACCA	hsa-miR-29a-3p / hsa-miR-29b-3p / hsa-miR-29c-3p	0.009408396	0.097031526	2	IRS1	LPL
UCACAGU	hsa-miR-27a-3p/hsa-miR-27b-3p	0.011872998	0.108997469	2	IRS1	LPL

microRNA	*p* value	Odd ratio	Number of interactions	Target Gene 1	Target Gene 2	
**Mienturnet Enrichment results using miRTarBase**						
hsa-miR-26b-5p	0.078148643	0.248805098	2	ITGB2	AGT	

## Discussion

**Fig. 3:**
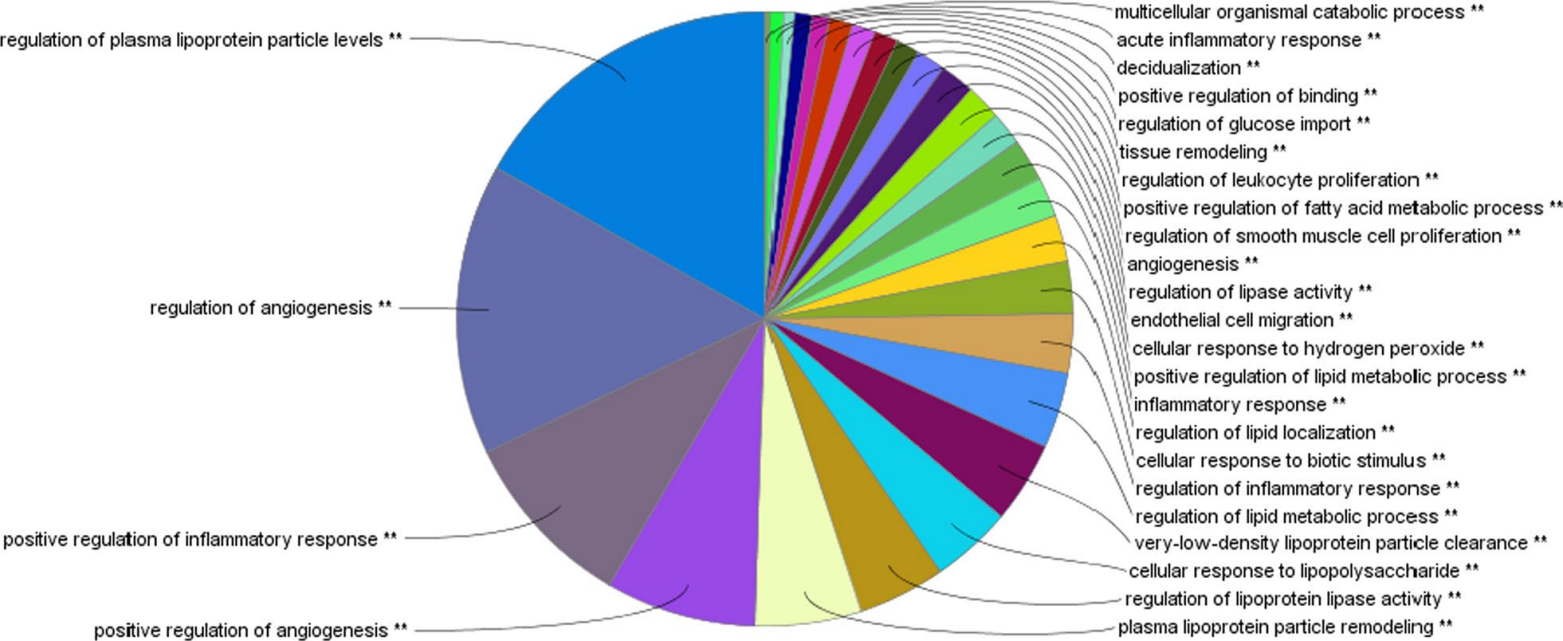
Pie chart depicting the 27 GO groups of cluster 3 generated by ClueGo. The area covered by each group represents the number of GO terms within each group. The most significant term of the group is labelled

Cardiovascular Disease (CVD) is the leading cause of death in the world and atherosclerosis being the major cause of CVD poses a serious health concern [68]. The pathophysiology of atherosclerosis involves the interplay of a number of inflammatory genes, lipid genes, also genes involved with diabetes and results in the manifestation of atherosclerotic plague [45, 63]. In the present study, in silico gene network analysis was used as a tool to prioritize the most influential gene/s among all the atherosclerotic genes that have key regulatory role in the pathogenesis of atherosclerosis. The reliability of in silico gene network analysis to predict influencial genes was elucidated by Baishya et al. in their recent publication [11]. For this study, 988 atherosclerotic genes were obtained from NCBI database; these genes were used to construct PPI using STRING. Subsequently, cluster analysis, topological parameters analysis and functional enrichment analysis helped in identifying 4 genes viz., AGT (Gene ID: 183), LPL (Gene ID: 4023), ITGB2 (Gene ID: 3689), and IRS1(Gene ID: 3667) from cluster 3 that may be considered to be most influential in the pathogenesis of atherosclerosis. Based on the MCODE score and functional enrichment analysis, cluster 3 was selected. Further on the basis of degree of nodes, betweenness centrality, and average shortest path length the genes having ≥ 100 degree were selected. The lists of selected genes are tabulated in Table 3. A pie diagram depicting the GO groups of cluster 3 is represented in Fig. 3

**Table 3:** List of selected genes

Name	MCODE Cluster	MCODE-Score	Avg. shortest pathlength	Betweeness centrality	Degree
AGT	["Cluster 3"]	27.08641975	1.91618829	0.00803355	161
LPL	["Cluster 3"]	27	2.00918485	0.0040675	113
ITGB2	["Cluster 3"]	25.69500675	2.06429392	0.00339199	104
IRS1	["Cluster 3"]	24.83599419	1.9793341	0.00162085	100

AGT encodes for angiotensinogen and is found to be associated with 111 GO terms viz., angiogenesis, regulation of lipase activity, vasoconstriction, regulation of blood pressure, regulation of ion homeostasis, renal system process, etc. involved in different GO groups. The list consisting of GO terms is tabulated in Additional file 7: Table S7a. Primarily, angiotensinogen is produced by liver and is the precursor of other angiotensins [85]. Angiotensin is a component of Renin–Angiotensin–Aldosterone (RAAS) system or Renin-Angiotensin system (RAS) and has a key implication on cardiovascular, renal, neural, endothelial system [66, 85]. When the sodium concentration in kidney is low, renin is secreted. It then acts at the 10N-terminal amino acid of angiotensinogen (AGT) to produce Angiotensin I (AngI), further angiotensin converting enzyme (ACE) catalyses the conversion of AngI to Angiotensin II (AngII) [29]. AngII and AngI are also catalyzed by ACE2 to produce Angiotensin 1–7. In systemic arterioles, AngII binds to Angiotensin II type 1 receptor (AT1) and Angiotensin II type 2 receptor (AT2) and acts as vasoconstrictor by enhancing fluid intake and increasing blood pressure. Whereas Ang1-7 act on Mas receptor and results in the synthesis of nitric oxide and prostaglandin and thereby causes vasodialation [29, 36, 85]. AngII stimulates the release of aldosterone and antidiuretic hormone which activates sodium reabsorption, potassium excretion, and water reabsorption in kidney respectively to maintain the blood volume and pressure [29]. AngII is however responsible to elicit atherosclerosis by recruiting monocytes, promoting macrophages lipid peroxidation and increasing oxidative stress. Hong Lu et al. found that when drugs that inhibit Renin were administered to LDL receptor deficient mice the progression of atherosclerosis was prevented by inhibiting the production of AngI [42]. Daugherty et al. have found that when AngII was infused in APOE deficient mice, the progression of atherosclerotic lesion was increased by influencing sheer stress in the artery, activation of monocytes and monocyte chemoattractant Protein-1 [22]. AngII causes oxidation of Low Density Lipoprotein (LDL) which in turn is taken up by the receptors of macrophages and leads to the formation of atherosclerotic lesion. Studies have shown that the inhibition of binding of AngII to ATI receptor can prevent the development of atherosclerosis [23, 37].

Also,when renin acts at the 10N-terminal amino acid of AGT, only 2% of it produces AngI but the remaining 98% of AGT known as des(AngI)AGT has independent role to perform in angiogenesis. In a study it was seen that synthesis of low concentration of AGT reduces body weight gain and liver steatosis which is contrast to AngII dependent function but the underlying mechanism is not clear. Thus, AngII independent AGT functions can be proved beneficial to hypertensive obesed individual. Also, when AGT was inhibited, the blood pressure and the development of atherosclerotic lesion was decreased, this is similar to the finding where when AngII was inhibited the blood pressure and development of atherosclerotic lesion was reduced. However, owing to the role in embryonic development of RAS system, the complete deletion of major RAS system can lead to low neonatal survival rate [22, 43].

LPL encodes for the enzyme lipoprotein lipase. It has been found that it is associated with 41 GO terms viz., inflammatory response, plasma lipoprotein particle remodeling, cholesterol homeostasis, regulation of macrophage derived foam cell differentiation, etc. involved in different GO groups. All are listed in the Additional file 7: Table S7b. LPL acts as a rate limiting enzyme by hydrolyzing the triglyceride present on Very Low Density Lipoprotein (VLDL) and chylomicron to free fatty acid and glycerol. The fatty acid so formed are taken up by tissues and acts as an energy source for cardiac muscle [59]. Dysfunction of LPL system can lead to the manifestation of atherosclerosis, chylomicronemia, dyslipidemia, obesity [50]. The LPL system has 3 important components: LPL, Apolipoprotein CII, glycosylphosphatidylinositol-anchored high density lipoprotein-binding protein 1 (GPIHBP1). Any anomality in these components can lead to hypertriglyceridemia [54]. Experiment on homozygous knockout mice has led to the development of hypertriglyceridemia, an increased level of VLDL and reduced adipose tissue store because triglycerides could not be hydrolysed and thus fatty acids could not be stored in adipose tissue [79]. *Invitro* study has revealed that LPL has differential role in expression of inflammatory genes that play a key role in atherosclerosis, it was seen that LPL had the ability to suppress TNFα-induced gene expression by inhibiting NF-B activation and induced IFNγ gene expression by activating STAT1 pathway [38]. In macrophage of LPL knockout mice, the plasma LPL and lipoprotein remained unchanged, cholesterol ester formation and intracellular triglyceride level was reduced and thus interfere with the development of atherosclerosis [8, 71]. There is also evidence that with the increase in the level of LPL, monocyte derived macrophage activation is triggered and the activator of LPL, APOC2 is also increased. These macrophages get laden with lipid to form foam cells which a vital step in the progress of atherosclerosis [51].

ITGB2 encodes for beta 2 intrigrin protein, it was found that it is associated with 19 GO terms viz., angiogenesis, inflammatory response, nitric oxide metabolic process, etc. involved in different GO groups. (Additional file 7: Table S7c). ITGB2 is transmembrane adhesion and signaling receptor expressed exclusively on leukocytes and extracellular vesicles. It has two sub units: a β chain (CD18) and one of the α chains-CD11a (LFA-1), CD11b (Mac-1), CD11c (p150, 95), or CD11d. It aids in the adhesion of leukocytes and its migration into the intima [25, 27, 52]. Once in the intima it triggers different key inflammatory molecules and immune mediators like ICAM1, VCAM1 thereby activating endothetial cells and stimulate macrophages to take up modified lipoproteins. Thus, aids in the process of atherosclerosis [25]. A study has shown that in CD18 knockout mice, the manifestation of atherosclerosis was prevented and also increased the capacity of macrophages to take up more modified lipoproteins and apoptotic cells. Thus, exhibiting a direct relationship of ITGB2 with atherosclerosis [25]. Another study on CD11d and APOE knockout mice revealed that CD11d has a proatherogenic role because the absence of CD11d has reduced the formation foam cells, proinflamatory cytokines which are the key steps in atherosclerosis [7]. An investigation by Huaizhu Wu et al. was done to determine the role of CD11c in hypercholesterolemia and atherogenic lesion and it was found that on APOE double deficiency hypercholesteromic mice the expression of CD11c was increased and the number of circulating monocytes were increased compared to wild type APOE double deficiency normal fed mice. And deficiency of CD11c on APOE double deficiency mice reduces the firm attachment of monocytes to VCAM1, P Selectin which are proinflamatory cytokine for the development of atherosclerosis [80].

IRS1 encodes for insulin receptor substrate 1 and is found to be associated with 40 GO terms viz., regulation of lipid metabolic process, regulation of glucose import, regulation of fatty acid metabolic process, etc. involved in different GO groups. All the GO terms are listed in the Additional file 7: Table S7d. IRS1 is a member of Insulin receptor family, which is a cytoplasmic adapter protein. IRS1 is mainly associated with glucose metabolism; in an experiment it was found that ubiquitinization and degradation of IRS1 resulted in the immobilization of GLUT4 transporter in adipocytes and impair its glucose uptake potency [47], 78. A similar result was found in another experiment that involved IRS1 knockout mice lacking hepatic IRS2, it was observed that leptin, IGF1 levels was decreased, glucose tolerance ability was impaired and resulted in hyperglycemia [24]. The development of hyperglycemic condition due to the degradation of IRS1 causes nonenzymatic glycosylation of lipids and proteins, and eventually triggers oxidative stress and activates protein kinase C (PKC). These lead to the modification of LDL and interferes with the LDL-Receptor mediated clearance of LDL, that further triggers the activation of monocyte derived macrophages to generate foam cells and thereby causing atherosclerosis [5]. In an experiment it was found that in IRS1 knockout mice during the hyperinsulinemic-euglycemic clamp condition, the plasma glycerol level and plasma free fatty acid were greatly reduced as compared to IRS2 deficient and wild type mice suggesting that IRS1 has more lipolysis suppressing potential than IRS2 [60]. Also, Sterol regulatory element binding protein 1c (SREBP1c) regulate the process of lipid metabolism by modulating many lipolytic gene expression. It was observed that the expression of the SREBP-1c gene and glucokinase gene were reduced when primary hepatocyte culture was infected with dominant-negative mutant of PI 3-kinase. Thus, IRS1- phosphatidylinositol (PI) 3-kinase pathway plays an important role in insulin metabolism by regulating the expression of SREBP-1c [49].

### Predicted atherosclerotic genes as druggable target

Based on literature mining and database search it was found that drugs like eplerenone, cilazapril, eprosartan are available that targets the renin, ACE, AT1 respectively of RAAS system by blocking their activity [87] (https://go.drugbank.com/, accessed on 12/02/2021). However to the best of our knowledge, no reports have been found that assert AGT as drug target of any synthesized drug. Since AGT is the only precursor for the derivation of the entire angiotensin family, targeting this could be proven to be effective. LPL is associated with the hydrolysis triglyerides and alteration in the normal physiology can lead to hypertriglyceridemia. Glycyrrhizic Acid a natural compound of licorice plants has been found to reduce the level of LPL expression (Yoke Yin, So Ha, & Abdul Kadir, 2010). However, no synthesized drug has been found which acts on LPL as a target. ITGB2 that encodes Beta 2 Integrins are exploited as drug target for autoimmune diseases. A drug BMS-587101 blocks CD11a activity and is known to prevent inflammation [14]. Thus, this can be a potential drug target for atherosclerosis which is an inflammation mediated pathogenesis. A natural compound Carainterol A extracted from *Caragana intermedia* was found to increase the level of IRS1, therapeutic intervention mediated through IRS1 was considered to be a novel approach in treating insulin resistance, as discussed previously insulin sensitivity is associated with hyperglycemic condition, hence IRS1 can also be targeted for treating atherosclerosis [46]. Thus, it was evident from the literature mining and database search that all the 4 genes can be treated as a potential drug target to mitigate the glitches of atherosclerosis.

### miRNA as a potential therapeutic

miRNAs are short, non-coding nucleotides that bind with mRNAs and regulate the expressions of proteins. Thus, altering the expression of miRNA can influence the pathophysiology of any disease. *In-silico *in vitro and/or in vivo approaches have been successfully employed in establishing miRNA as potential drug target. The initiation of clinical trial of Miravirsen, a miRNA-122 repressor and RG-101 for the treatment of hepatitis C virus have set up the beginning of the upcoming era of miRNA based drugs [17, 31]. Role of miRNAs in the pathophysiology of cardiovascular disease have been studied extensively and are regarded as potential drug targets [18, 62, 93].

miRNA-Target prediction revealed miRNA families that target IRS1 and LPL are hsa-miR-27a-3p/hsa-miR-27b-3p and hsa-miR-29a-3p/hsa-miR-29b-3p/hsa-miR-29c-3p, and IRS1 and ITGB2 are targeted by hsa-miR-26b-5p. A number of papers reported that miRNAs are associated with different cardiovascular complications [62, 93]. The reduced level of miR-27a, miR-29a, and miR-26b cause heart failure [93]. Down regulation of miR-26 is associated with atrial fibrillation, thus overexpression reduces the susceptibility of atrial fibrillation [44]. The over-expression of miR-26a/b causes alteration in the cholesterol synthesis [53]. When miRNA expression profiling was conducted in atherosclerotic model, exhibiting hypertension and hyperlipidemia, there was a significant increase in the expression of miR-26b along with other miRNAs [2]. Also, down regulation of miR‐26a triggers angiotensin II‐induced fibrogenesis in cardiac fibroblasts resulting in the occurrence of hypertensive myocardial fibrosis [90, 92]. The upregulation of miR-27b causes cardiac hypertrophy and its suppression cause inhibition of hypertropic cell growth [77]. miR-27a up-regulation suppresses the differentiation of adipocytes which are the primary sites of cholesterol metabolism [1, 61], up-regulation of miR-27b is reported to be associated with dyslipidemia and atherosclerosis [75]. miR-29a is known to target IRS1 and alter the Akt/GSK-3 pathway and glucose uptake, it also impairs endothelial cells function; miR-29c is suspected to have antiangiogenic effect  [91]. miR-29 regulates the genes that are responsible for lipid metabolism, miR-29a up-regulation enhances the chances of insulin resistence [81]. It was observed that the expression of miR-29 was down-regulated in the area of fibrotic lessions that elucidates the role of miR-29 in causing cardiac fibrosis [74]. A drug called Miragen that targets miR-29 is already in active phase 1 trial to treat keloid and scar tissue [31]. Apart from it, other drugs that target different miRNAs are also in clinical trials [89]. Thus, it could be understood from the above discussion that the regulatory role of miRNAs are very critical and hence they are nowadays considered as new generation therapeutics to combat various metabolic diseases.

## Conclusion

Owing to the seriousness of atherosclerosis the identification of the key genes that play influential role in causing this condition is the need of the hour. From the vast array of genes that are associated with this condition, AGT, LPL, ITGB2, IRS were identified to play vital role in the pathogenesis of atherosclerosis. AGT is the primary precursor of angiotensins and aid in the regulation of angiogenesis and blood pressure, LPL hydrolyzes triglyceride of VLDL and chylomicron. ITGB2 facilitates the adhesion and migration of leukocytes and generate inflammatory response. IRS1 is primarily associated with glucose metabolism and hyperglycemia. However, these genes are regulated by miRNAs: miR-26, miR-27, and miR-29 families. miRNAs are non-coding nucleotides that are associated with various diseases by regulating the expression of respective genes at the post transcriptional level. Hence, either these genes or the miRNAs interacting with these genes could be regarded as potential targets for therapeutic drug intervention of atherosclerosis. However, translational validation is essential for the final establishment of these genes as possible atherosclerotic drug target.

## Supplementary Information

**Additional file 1**. List of genes involved in atherosclerosis.

**Additional file 2**. Protein protein interaction  at different confidence score.

**Additional file 3**. Topological parameter of all the clustered nodes obtained by MCODE.

**Additional file 4**. List of all GO terms of 6 clusters obtained from ClueGo.

**Additional file 5**. Topological parameter of nodes obtained by MIENTURNET.

**Additional file 6**. Disease ontology of miRNA obtained by MIENTURNET.

**Additional file 7**. List of biological processes mediated by 4 genes obtained by ClueGo.

**Additional file 8**. Clusters generated after MCODE analysis.

## Data Availability

The datasets analysed during the current study are available and in the NCBI repository and can be downloaded, (https://www.ncbi.nlm.nih.gov/gene, and accessed on 22/12/2020). The accession numbers are provided in the Additional file 1.

## Abbreviations

PPI: Protein–protein interaction
CVD: Cardiovascular disease
GO: Gene ontology
LPL: Lipoprotein lipase
AGT: Angiotensinogen
IRS1: Insulin receptor substrate
ITGB2: Beta 2 integrin
ACE: Angiotensin converting enzyme
AT1: Angiotensin II type 1 receptor
SREBP: Sterol regulatory element-binding transcription factor
PKC: Protein kinase C
RAAS: Renin- angiotensin-aldosterone system
IGF: Insulin-like growth factor
VCAM: Vascular cell adhesion molecule
APOE: Apolipoprotein E
ICAM: Intercellular adhesion molecule
APOC2: Apolipoprotein C2
STAT: Signal transducer and activator of transcription
IFN: Interferon
TNF: Tumor necrotic factor
VLDL: Very low density lipoprotein
Ang: Angiotensin
LDL: Low density lipoprotein
ABCA1: ATP-binding cassette transporter
LCAT: Lecithin–cholesterol acyltransferase
CETP: Cholesteryl ester transfer protein
PPAR: Peroxisome proliferator-activated receptors
LXR: Liver X receptor
SR-A: Scavenger receptor A
FoxO1: Forkhead box protein O1
PCSK9: Proproteinconvertasesubtilisin/kexin type 9
MCP: Monocyte chemoattractantprotein
M-CSF: Macrophage colony-stimulating factor
IL: Interleukin
GPIHBP1: Glycosylphosphatidylinositol-anchored high density lipoprotein-binding protein 1
