# Supplementary material for: Network analysis of atherosclerotic genes elucidates druggable targets
Source: BMC Med Genomics. 2022 Mar 3;15:42. doi: 10.1186/s12920-022-01195-y (PMC8893053; doi:10.1186/s12920-022-01195-y)
Supplement: Supplementary file 1 — Additional file 1. List of genes involved in atherosclerosis. [file 12920_2022_1195_MOESM1_ESM.docx]

**Additional File 1**

**Table 1: List of genes involved in atherosclerosis**

| **Sl No.** | **GeneID** | **Symbol** | **Description** |
| --- | --- | --- | --- |
|  | 1029 | CDKN2A | cyclin dependent kinase inhibitor 2A |
|  | 348 | APOE | apolipoprotein E |
|  | 3084 | NRG1 | neuregulin 1 |
|  | 1030 | CDKN2B | cyclin dependent kinase inhibitor 2B |
|  | 335 | APOA1 | apolipoprotein A1 |
|  | 100048912 | CDKN2B-AS1 | CDKN2B antisense RNA 1 |
|  | 338 | APOB | apolipoprotein B |
|  | 3949 | LDLR | low density lipoprotein receptor |
|  | 28 | ABO | ABO, alpha 1-3-N-acetylgalactosaminyltransferase and alpha 1-3-galactosyltransferase |
|  | 7124 | TNF | tumor necrosis factor |
|  | 19 | ABCA1 | ATP binding cassette subfamily A member 1 |
|  | 1071 | CETP | cholesteryl ester transfer protein |
|  | 7157 | TP53 | tumor protein p53 |
|  | 7422 | VEGFA | vascular endothelial growth factor A |
|  | 596 | BCL2 | BCL2 apoptosis regulator |
|  | 3569 | IL6 | interleukin 6 |
|  | 255738 | PCSK9 | proprotein convertase subtilisin/kexin type 9 |
|  | 7040 | TGFB1 | transforming growth factor beta 1 |
|  | 4023 | LPL | lipoprotein lipase |
|  | 4524 | MTHFR | methylenetetrahydrofolate reductase |
|  | 3586 | IL10 | interleukin 10 |
|  | 4318 | MMP9 | matrix metallopeptidase 9 |
|  | 9370 | ADIPOQ | adiponectin, C1Q and collagen domain containing |
|  | 1401 | CRP | C-reactive protein |
|  | 2646 | GCKR | glucokinase regulator |
|  | 6272 | SORT1 | sortilin 1 |
|  | 116519 | APOA5 | apolipoprotein A5 |
|  | 7099 | TLR4 | toll like receptor 4 |
|  | 4846 | NOS3 | nitric oxide synthase 3 |
|  | 5468 | PPARG | peroxisome proliferator activated receptor gamma |
|  | 1636 | ACE | angiotensin I converting enzyme |
|  | 5265 | SERPINA1 | serpin family A member 1 |
|  | 4790 | NFKB1 | nuclear factor kappa B subunit 1 |
|  | 4255 | MGMT | O-6-methylguanine-DNA methyltransferase |
|  | 345 | APOC3 | apolipoprotein C3 |
|  | 3479 | IGF1 | insulin like growth factor 1 |
|  | 7139 | TNNT2 | troponin T2, cardiac type |
|  | 5444 | PON1 | paraoxonase 1 |
|  | 6347 | CCL2 | C-C motif chemokine ligand 2 |
|  | 5743 | PTGS2 | prostaglandin-endoperoxide synthase 2 |
|  | 3990 | LIPC | lipase C, hepatic type |
|  | 3605 | IL17A | interleukin 17A |
|  | 207 | AKT1 | AKT serine/threonine kinase 1 |
|  | 846 | CASR | calcium sensing receptor |
|  | 3553 | IL1B | interleukin 1 beta |
|  | 2099 | ESR1 | estrogen receptor 1 |
|  | 3091 | HIF1A | hypoxia inducible factor 1 subunit alpha |
|  | 3240 | HP | haptoglobin |
|  | 3162 | HMOX1 | heme oxygenase 1 |
|  | 3383 | ICAM1 | intercellular adhesion molecule 1 |
|  | 23411 | SIRT1 | sirtuin 1 |
|  | 7128 | TNFAIP3 | TNF alpha induced protein 3 |
|  | 5624 | PROC | protein C, inactivator of coagulation factors Va and VIIIa |
|  | 3952 | LEP | leptin |
|  | 177 | AGER | advanced glycosylation end-product specific receptor |
|  | 672 | BRCA1 | BRCA1 DNA repair associated |
|  | 3606 | IL18 | interleukin 18 |
|  | 4313 | MMP2 | matrix metallopeptidase 2 |
|  | 6774 | STAT3 | signal transducer and activator of transcription 3 |
|  | 7097 | TLR2 | toll like receptor 2 |
|  | 6696 | SPP1 | secreted phosphoprotein 1 |
|  | 7421 | VDR | vitamin D receptor |
|  | 5054 | SERPINE1 | serpin family E member 1 |
|  | 3576 | CXCL8 | C-X-C motif chemokine ligand 8 |
|  | 2638 | GC | GC vitamin D binding protein |
|  | 7015 | TERT | telomerase reverse transcriptase |
|  | 4879 | NPPB | natriuretic peptide B |
|  | 5243 | ABCB1 | ATP binding cassette subfamily B member 1 |
|  | 7852 | CXCR4 | C-X-C motif chemokine receptor 4 |
|  | 367 | AR | androgen receptor |
|  | 1906 | EDN1 | endothelin 1 |
|  | 3123 | HLA-DRB1 | major histocompatibility complex, class II, DR beta 1 |
|  | 627 | BDNF | brain derived neurotrophic factor |
|  | 5594 | MAPK1 | mitogen-activated protein kinase 1 |
|  | 4982 | TNFRSF11B | TNF receptor superfamily member 11b |
|  | 1312 | COMT | catechol-O-methyltransferase |
|  | 2475 | MTOR | mechanistic target of rapamycin kinase |
|  | 929 | CD14 | CD14 molecule |
|  | 114548 | NLRP3 | NLR family pyrin domain containing 3 |
|  | 4771 | NF2 | neurofibromin 2 |
|  | 5728 | PTEN | phosphatase and tensin homolog |
|  | 2944 | GSTM1 | glutathione S-transferase mu 1 |
|  | 1499 | CTNNB1 | catenin beta 1 |
|  | 7450 | VWF | von Willebrand factor |
|  | 57818 | G6PC2 | glucose-6-phosphatase catalytic subunit 2 |
|  | 6387 | CXCL12 | C-X-C motif chemokine ligand 12 |
|  | 4353 | MPO | myeloperoxidase |
|  | 6532 | SLC6A4 | solute carrier family 6 member 4 |
|  | 3146 | HMGB1 | high mobility group box 1 |
|  | 2952 | GSTT1 | glutathione S-transferase theta 1 |
|  | 56729 | RETN | resistin |
|  | 8074 | FGF23 | fibroblast growth factor 23 |
|  | 183 | AGT | angiotensinogen |
|  | 6647 | SOD1 | superoxide dismutase 1 |
|  | 406991 | MIR21 | microRNA 21 |
|  | 5360 | PLTP | phospholipid transfer protein |
|  | 999 | CDH1 | cadherin 1 |
|  | 5581 | PRKCE | protein kinase C epsilon |
|  | 2161 | F12 | coagulation factor XII |
|  | 5196 | PF4 | platelet factor 4 |
|  | 5649 | RELN | reelin |
|  | 406947 | MIR155 | microRNA 155 |
|  | 3458 | IFNG | interferon gamma |
|  | 3958 | LGALS3 | galectin 3 |
|  | 11173 | ADAMTS7 | ADAM metallopeptidase with thrombospondin type 1 motif 7 |
|  | 51738 | GHRL | ghrelin and obestatin prepropeptide |
|  | 185 | AGTR1 | angiotensin II receptor type 1 |
|  | 3717 | JAK2 | Janus kinase 2 |
|  | 2152 | F3 | coagulation factor III, tissue factor |
|  | 3630 | INS | insulin |
|  | 1234 | CCR5 | C-C motif chemokine receptor 5 |
|  | 920 | CD4 | CD4 molecule |
|  | 4780 | NFE2L2 | nuclear factor, erythroid 2 like 2 |
|  | 10544 | PROCR | protein C receptor |
|  | 5806 | PTX3 | pentraxin 3 |
|  | 2950 | GSTP1 | glutathione S-transferase pi 1 |
|  | 2147 | F2 | coagulation factor II, thrombin |
|  | 948 | CD36 | CD36 molecule |
|  | 4018 | LPA | lipoprotein(a) |
|  | 4312 | MMP1 | matrix metallopeptidase 1 |
|  | 154 | ADRB2 | adrenoceptor beta 2 |
|  | 10563 | CXCL13 | C-X-C motif chemokine ligand 13 |
|  | 7412 | VCAM1 | vascular cell adhesion molecule 1 |
|  | 960 | CD44 | CD44 molecule (Indian blood group) |
|  | 4851 | NOTCH1 | notch receptor 1 |
|  | 406938 | MIR146A | microRNA 146a |
|  | 2100 | ESR2 | estrogen receptor 2 |
|  | 2153 | F5 | coagulation factor V |
|  | 4000 | LMNA | lamin A/C |
|  | 959 | CD40LG | CD40 ligand |
|  | 1557 | CYP2C19 | cytochrome P450 family 2 subfamily C member 19 |
|  | 1559 | CYP2C9 | cytochrome P450 family 2 subfamily C member 9 |
|  | 8743 | TNFSF10 | TNF superfamily member 10 |
|  | 5820 | PVT1 | Pvt1 oncogene |
|  | 3934 | LCN2 | lipocalin 2 |
|  | 50943 | FOXP3 | forkhead box P3 |
|  | 3690 | ITGB3 | integrin subunit beta 3 |
|  | 1471 | CST3 | cystatin C |
|  | 337 | APOA4 | apolipoprotein A4 |
|  | 4314 | MMP3 | matrix metallopeptidase 3 |
|  | 4544 | MTNR1B | melatonin receptor 1B |
|  | 4282 | MIF | macrophage migration inhibitory factor |
|  | 3077 | HFE | homeostatic iron regulator |
|  | 3815 | KIT | KIT proto-oncogene, receptor tyrosine kinase |
|  | 2908 | NR3C1 | nuclear receptor subfamily 3 group C member 1 |
|  | 3480 | IGF1R | insulin like growth factor 1 receptor |
|  | 8600 | TNFSF11 | TNF superfamily member 11 |
|  | 213 | ALB | albumin |
|  | 9518 | GDF15 | growth differentiation factor 15 |
|  | 341 | APOC1 | apolipoprotein C1 |
|  | 5970 | RELA | RELA proto-oncogene, NF-kB subunit |
|  | 3082 | HGF | hepatocyte growth factor |
|  | 3992 | FADS1 | fatty acid desaturase 1 |
|  | 3557 | IL1RN | interleukin 1 receptor antagonist |
|  | 3688 | ITGB1 | integrin subunit beta 1 |
|  | 64127 | NOD2 | nucleotide binding oligomerization domain containing 2 |
|  | 2146 | EZH2 | enhancer of zeste 2 polycomb repressive complex 2 subunit |
|  | 836 | CASP3 | caspase 3 |
|  | 27122 | DKK3 | dickkopf WNT signaling pathway inhibitor 3 |
|  | 10499 | NCOA2 | nuclear receptor coactivator 2 |
|  | 6374 | CXCL5 | C-X-C motif chemokine ligand 5 |
|  | 7941 | PLA2G7 | phospholipase A2 group VII |
|  | 6648 | SOD2 | superoxide dismutase 2 |
|  | 4153 | MBL2 | mannose binding lectin 2 |
|  | 4973 | OLR1 | oxidized low density lipoprotein receptor 1 |
|  | 1027 | CDKN1B | cyclin dependent kinase inhibitor 1B |
|  | 6714 | SRC | SRC proto-oncogene, non-receptor tyrosine kinase |
|  | 5465 | PPARA | peroxisome proliferator activated receptor alpha |
|  | 5329 | PLAUR | plasminogen activator, urokinase receptor |
|  | 10891 | PPARGC1A | PPARG coactivator 1 alpha |
|  | 6772 | STAT1 | signal transducer and activator of transcription 1 |
|  | 9415 | FADS2 | fatty acid desaturase 2 |
|  | 6401 | SELE | selectin E |
|  | 4928 | NUP98 | nucleoporin 98 and 96 precursor |
|  | 90865 | IL33 | interleukin 33 |
|  | 9365 | KL | klotho |
|  | 5595 | MAPK3 | mitogen-activated protein kinase 3 |
|  | 387 | RHOA | ras homolog family member A |
|  | 2167 | FABP4 | fatty acid binding protein 4 |
|  | 3791 | KDR | kinase insert domain receptor |
|  | 3725 | JUN | Jun proto-oncogene, AP-1 transcription factor subunit |
|  | 1535 | CYBA | cytochrome b-245 alpha chain |
|  | 10135 | NAMPT | nicotinamide phosphoribosyltransferase |
|  | 3075 | CFH | complement factor H |
|  | 949 | SCARB1 | scavenger receptor class B member 1 |
|  | 3953 | LEPR | leptin receptor |
|  | 1952 | CELSR2 | cadherin EGF LAG seven-pass G-type receptor 2 |
|  | 3039 | HBA1 | hemoglobin subunit alpha 1 |
|  | 8650 | NUMB | NUMB endocytic adaptor protein |
|  | 1588 | CYP19A1 | cytochrome P450 family 19 subfamily A member 1 |
|  | 958 | CD40 | CD40 molecule |
|  | 5950 | RBP4 | retinol binding protein 4 |
|  | 3329 | HSPD1 | heat shock protein family D (Hsp60) member 1 |
|  | 2321 | FLT1 | fms related receptor tyrosine kinase 1 |
|  | 1116 | CHI3L1 | chitinase 3 like 1 |
|  | 6280 | S100A9 | S100 calcium binding protein A9 |
|  | 2157 | F8 | coagulation factor VIII |
|  | 4843 | NOS2 | nitric oxide synthase 2 |
|  | 3552 | IL1A | interleukin 1 alpha |
|  | 2678 | GGT1 | gamma-glutamyltransferase 1 |
|  | 1803 | DPP4 | dipeptidyl peptidase 4 |
|  | 9314 | KLF4 | Kruppel like factor 4 |
|  | 3315 | HSPB1 | heat shock protein family B (small) member 1 |
|  | 2308 | FOXO1 | forkhead box O1 |
|  | 2335 | FN1 | fibronectin 1 |
|  | 7132 | TNFRSF1A | TNF receptor superfamily member 1A |
|  | 10 | NAT2 | N-acetyltransferase 2 |
|  | 5473 | PPBP | pro-platelet basic protein |
|  | 196 | AHR | aryl hydrocarbon receptor |
|  | 217 | ALDH2 | aldehyde dehydrogenase 2 family member |
|  | 729230 | CCR2 | C-C motif chemokine receptor 2 |
|  | 682 | BSG | basigin (Ok blood group) |
|  | 356 | FASLG | Fas ligand |
|  | 27329 | ANGPTL3 | angiopoietin like 3 |
|  | 1950 | EGF | epidermal growth factor |
|  | 6790 | AURKA | aurora kinase A |
|  | 2896 | GRN | granulin precursor |
|  | 59272 | ACE2 | angiotensin I converting enzyme 2 |
|  | 2697 | GJA1 | gap junction protein alpha 1 |
|  | 197 | AHSG | alpha 2-HS glycoprotein |
|  | 2067 | ERCC1 | ERCC excision repair 1, endonuclease non-catalytic subunit |
|  | 3481 | IGF2 | insulin like growth factor 2 |
|  | 6352 | CCL5 | C-C motif chemokine ligand 5 |
|  | 2309 | FOXO3 | forkhead box O3 |
|  | 3309 | HSPA5 | heat shock protein family A (Hsp70) member 5 |
|  | 3486 | IGFBP3 | insulin like growth factor binding protein 3 |
|  | 8842 | PROM1 | prominin 1 |
|  | 650 | BMP2 | bone morphogenetic protein 2 |
|  | 9021 | SOCS3 | suppressor of cytokine signaling 3 |
|  | 6288 | SAA1 | serum amyloid A1 |
|  | 1524 | CX3CR1 | C-X3-C motif chemokine receptor 1 |
|  | 718 | C3 | complement C3 |
|  | 407040 | MIR34A | microRNA 34a |
|  | 153090 | DAB2IP | DAB2 interacting protein |
|  | 26191 | PTPN22 | protein tyrosine phosphatase non-receptor type 22 |
|  | 366 | AQP9 | aquaporin 9 |
|  | 7076 | TIMP1 | TIMP metallopeptidase inhibitor 1 |
|  | 22943 | DKK1 | dickkopf WNT signaling pathway inhibitor 1 |
|  | 5069 | PAPPA | pappalysin 1 |
|  | 3559 | IL2RA | interleukin 2 receptor subunit alpha |
|  | 3308 | HSPA4 | heat shock protein family A (Hsp70) member 4 |
|  | 4035 | LRP1 | LDL receptor related protein 1 |
|  | 6403 | SELP | selectin P |
|  | 1786 | DNMT1 | DNA methyltransferase 1 |
|  | 8658 | TNKS | tankyrase |
|  | 3667 | IRS1 | insulin receptor substrate 1 |
|  | 5328 | PLAU | plasminogen activator, urokinase |
|  | 11093 | ADAMTS13 | ADAM metallopeptidase with thrombospondin type 1 motif 13 |
|  | 26291 | FGF21 | fibroblast growth factor 21 |
|  | 2155 | F7 | coagulation factor VII |
|  | 10855 | HPSE | heparanase |
|  | 2214 | FCGR3A | Fc fragment of IgG receptor IIIa |
|  | 470 | ATHS | atherosclerosis susceptibility (lipoprotein associated) |
|  | 796 | CALCA | calcitonin related polypeptide alpha |
|  | 5228 | PGF | placental growth factor |
|  | 378938 | MALAT1 | metastasis associated lung adenocarcinoma transcript 1 |
|  | 1268 | CNR1 | cannabinoid receptor 1 |
|  | 4049 | LTA | lymphotoxin alpha |
|  | 3600 | IL15 | interleukin 15 |
|  | 5027 | P2RX7 | purinergic receptor P2X 7 |
|  | 7431 | VIM | vimentin |
|  | 6850 | SYK | spleen associated tyrosine kinase |
|  | 285 | ANGPT2 | angiopoietin 2 |
|  | 4323 | MMP14 | matrix metallopeptidase 14 |
|  | 3620 | IDO1 | indoleamine 2,3-dioxygenase 1 |
|  | 567 | B2M | beta-2-microglobulin |
|  | 5176 | SERPINF1 | serpin family F member 1 |
|  | 3689 | ITGB2 | integrin subunit beta 2 |
|  | 406937 | MIR145 | microRNA 145 |
|  | 4317 | MMP8 | matrix metallopeptidase 8 |
|  | 57817 | HAMP | hepcidin antimicrobial peptide |
|  | 6868 | ADAM17 | ADAM metallopeptidase domain 17 |
|  | 23405 | DICER1 | dicer 1, ribonuclease III |
|  | 2022 | ENG | endoglin |
|  | 998 | CDC42 | cell division cycle 42 |
|  | 6093 | ROCK1 | Rho associated coiled-coil containing protein kinase 1 |
|  | 2212 | FCGR2A | Fc fragment of IgG receptor IIa |
|  | 7052 | TGM2 | transglutaminase 2 |
|  | 10673 | TNFSF13B | TNF superfamily member 13b |
|  | 4644 | MYO5A | myosin VA |
|  | 3685 | ITGAV | integrin subunit alpha V |
|  | 6615 | SNAI1 | snail family transcriptional repressor 1 |
|  | 7056 | THBD | thrombomodulin |
|  | 6794 | STK11 | serine/threonine kinase 11 |
|  | 7840 | ALMS1 | ALMS1 centrosome and basal body associated protein |
|  | 6927 | HNF1A | HNF1 homeobox A |
|  | 947 | CD34 | CD34 molecule |
|  | 268 | AMH | anti-Mullerian hormone |
|  | 285440 | CYP4V2 | cytochrome P450 family 4 subfamily V member 2 |
|  | 5741 | PTH | parathyroid hormone |
|  | 7295 | TXN | thioredoxin |
|  | 1994 | ELAVL1 | ELAV like RNA binding protein 1 |
|  | 7163 | TPD52 | tumor protein D52 |
|  | 4854 | NOTCH3 | notch receptor 3 |
|  | 5478 | PPIA | peptidylprolyl isomerase A |
|  | 3956 | LGALS1 | galectin 1 |
|  | 3570 | IL6R | interleukin 6 receptor |
|  | 463 | ZFHX3 | zinc finger homeobox 3 |
|  | 252995 | FNDC5 | fibronectin type III domain containing 5 |
|  | 80339 | PNPLA3 | patatin like phospholipase domain containing 3 |
|  | 3673 | ITGA2 | integrin subunit alpha 2 |
|  | 4316 | MMP7 | matrix metallopeptidase 7 |
|  | 820 | CAMP | cathelicidin antimicrobial peptide |
|  | 7424 | VEGFC | vascular endothelial growth factor C |
|  | 2069 | EREG | epiregulin |
|  | 7474 | WNT5A | Wnt family member 5A |
|  | 2784 | GNB3 | G protein subunit beta 3 |
|  | 6720 | SREBF1 | sterol regulatory element binding transcription factor 1 |
|  | 4852 | NPY | neuropeptide Y |
|  | 3818 | KLKB1 | kallikrein B1 |
|  | 6279 | S100A8 | S100 calcium binding protein A8 |
|  | 6376 | CX3CL1 | C-X3-C motif chemokine ligand 1 |
|  | 5159 | PDGFRB | platelet derived growth factor receptor beta |
|  | 1536 | CYBB | cytochrome b-245 beta chain |
|  | 133 | ADM | adrenomedullin |
|  | 11315 | PARK7 | Parkinsonism associated deglycase |
|  | 79001 | VKORC1 | vitamin K epoxide reductase complex subunit 1 |
|  | 7035 | TFPI | tissue factor pathway inhibitor |
|  | 7351 | UCP2 | uncoupling protein 2 |
|  | 5562 | PRKAA1 | protein kinase AMP-activated catalytic subunit alpha 1 |
|  | 6462 | SHBG | sex hormone binding globulin |
|  | 1585 | CYP11B2 | cytochrome P450 family 11 subfamily B member 2 |
|  | 5340 | PLG | plasminogen |
|  | 5058 | PAK1 | p21 (RAC1) activated kinase 1 |
|  | 652 | BMP4 | bone morphogenetic protein 4 |
|  | 50507 | NOX4 | NADPH oxidase 4 |
|  | 5327 | PLAT | plasminogen activator, tissue type |
|  | 8651 | SOCS1 | suppressor of cytokine signaling 1 |
|  | 864 | RUNX3 | RUNX family transcription factor 3 |
|  | 246 | ALOX15 | arachidonate 15-lipoxygenase |
|  | 346007 | EYS | eyes shut homolog |
|  | 2590 | GALNT2 | polypeptide N-acetylgalactosaminyltransferase 2 |
|  | 632 | BGLAP | bone gamma-carboxyglutamate protein |
|  | 240 | ALOX5 | arachidonate 5-lipoxygenase |
|  | 6263 | RYR3 | ryanodine receptor 3 |
|  | 102 | ADAM10 | ADAM metallopeptidase domain 10 |
|  | 3674 | ITGA2B | integrin subunit alpha 2b |
|  | 406906 | MIR122 | microRNA 122 |
|  | 847 | CAT | catalase |
|  | 155 | ADRB3 | adrenoceptor beta 3 |
|  | 358 | AQP1 | aquaporin 1 (Colton blood group) |
|  | 149233 | IL23R | interleukin 23 receptor |
|  | 2149 | F2R | coagulation factor II thrombin receptor |
|  | 3484 | IGFBP1 | insulin like growth factor binding protein 1 |
|  | 775 | CACNA1C | calcium voltage-gated channel subunit alpha1 C |
|  | 22914 | KLRK1 | killer cell lectin like receptor K1 |
|  | 50616 | IL22 | interleukin 22 |
|  | 3551 | IKBKB | inhibitor of nuclear factor kappa B kinase subunit beta |
|  | 551 | AVP | arginine vasopressin |
|  | 8862 | APLN | apelin |
|  | 5467 | PPARD | peroxisome proliferator activated receptor delta |
|  | 3931 | LCAT | lecithin-cholesterol acyltransferase |
|  | 84868 | HAVCR2 | hepatitis A virus cellular receptor 2 |
|  | 5320 | PLA2G2A | phospholipase A2 group IIA |
|  | 407006 | MIR221 | microRNA 221 |
|  | 4803 | NGF | nerve growth factor |
|  | 8856 | NR1I2 | nuclear receptor subfamily 1 group I member 2 |
|  | 54790 | TET2 | tet methylcytosine dioxygenase 2 |
|  | 7534 | YWHAZ | tyrosine 3-monooxygenase/tryptophan 5-monooxygenase activation protein zeta |
|  | 406992 | MIR210 | microRNA 210 |
|  | 3579 | CXCR2 | C-X-C motif chemokine receptor 2 |
|  | 2641 | GCG | glucagon |
|  | 8542 | APOL1 | apolipoprotein L1 |
|  | 5777 | PTPN6 | protein tyrosine phosphatase non-receptor type 6 |
|  | 50964 | SOST | sclerostin |
|  | 9332 | CD163 | CD163 molecule |
|  | 8742 | TNFSF12 | TNF superfamily member 12 |
|  | 406913 | MIR126 | microRNA 126 |
|  | 7329 | UBE2I | ubiquitin conjugating enzyme E2 I |
|  | 2852 | GPER1 | G protein-coupled estrogen receptor 1 |
|  | 3371 | TNC | tenascin C |
|  | 1665 | DHX15 | DEAH-box helicase 15 |
|  | 6557 | SLC12A1 | solute carrier family 12 member 1 |
|  | 3592 | IL12A | interleukin 12A |
|  | 7045 | TGFBI | transforming growth factor beta induced |
|  | 940 | CD28 | CD28 molecule |
|  | 3611 | ILK | integrin linked kinase |
|  | 407021 | MIR29A | microRNA 29a |
|  | 246778 | IL27 | interleukin 27 |
|  | 301 | ANXA1 | annexin A1 |
|  | 84876 | ORAI1 | ORAI calcium release-activated calcium modulator 1 |
|  | 6576 | SLC25A1 | solute carrier family 25 member 1 |
|  | 350 | APOH | apolipoprotein H |
|  | 5919 | RARRES2 | retinoic acid receptor responder 2 |
|  | 8829 | NRP1 | neuropilin 1 |
|  | 1969 | EPHA2 | EPH receptor A2 |
|  | 1113 | CHGA | chromogranin A |
|  | 1508 | CTSB | cathepsin B |
|  | 5788 | PTPRC | protein tyrosine phosphatase receptor type C |
|  | 2006 | ELN | elastin |
|  | 5175 | PECAM1 | platelet and endothelial cell adhesion molecule 1 |
|  | 3575 | IL7R | interleukin 7 receptor |
|  | 3716 | JAK1 | Janus kinase 1 |
|  | 51284 | TLR7 | toll like receptor 7 |
|  | 1509 | CTSD | cathepsin D |
|  | 2870 | GRK6 | G protein-coupled receptor kinase 6 |
|  | 3814 | KISS1 | KiSS-1 metastasis suppressor |
|  | 29119 | CTNNA3 | catenin alpha 3 |
|  | 7078 | TIMP3 | TIMP metallopeptidase inhibitor 3 |
|  | 406935 | MIR143 | microRNA 143 |
|  | 10062 | NR1H3 | nuclear receptor subfamily 1 group H member 3 |
|  | 2919 | CXCL1 | C-X-C motif chemokine ligand 1 |
|  | 55600 | ITLN1 | intelectin 1 |
|  | 1991 | ELANE | elastase, neutrophil expressed |
|  | 1272 | CNTN1 | contactin 1 |
|  | 1003 | CDH5 | cadherin 5 |
|  | 5335 | PLCG1 | phospholipase C gamma 1 |
|  | 4878 | NPPA | natriuretic peptide A |
|  | 3572 | IL6ST | interleukin 6 signal transducer |
|  | 1020 | CDK5 | cyclin dependent kinase 5 |
|  | 10630 | PDPN | podoplanin |
|  | 27178 | IL37 | interleukin 37 |
|  | 51561 | IL23A | interleukin 23 subunit alpha |
|  | 7186 | TRAF2 | TNF receptor associated factor 2 |
|  | 1025 | CDK9 | cyclin dependent kinase 9 |
|  | 1033 | CDKN3 | cyclin dependent kinase inhibitor 3 |
|  | 781 | CACNA2D1 | calcium voltage-gated channel auxiliary subunit alpha2delta 1 |
|  | 4067 | LYN | LYN proto-oncogene, Src family tyrosine kinase |
|  | 284 | ANGPT1 | angiopoietin 1 |
|  | 182 | JAG1 | jagged canonical Notch ligand 1 |
|  | 9619 | ABCG1 | ATP binding cassette subfamily G member 1 |
|  | 5144 | PDE4D | phosphodiesterase 4D |
|  | 2833 | CXCR3 | C-X-C motif chemokine receptor 3 |
|  | 308 | ANXA5 | annexin A5 |
|  | 4306 | NR3C2 | nuclear receptor subfamily 3 group C member 2 |
|  | 1361 | CPB2 | carboxypeptidase B2 |
|  | 6441 | SFTPD | surfactant protein D |
|  | 6010 | RHO | rhodopsin |
|  | 4092 | SMAD7 | SMAD family member 7 |
|  | 27165 | GLS2 | glutaminase 2 |
|  | 54210 | TREM1 | triggering receptor expressed on myeloid cells 1 |
|  | 5590 | PRKCZ | protein kinase C zeta |
|  | 966 | CD59 | CD59 molecule (CD59 blood group) |
|  | 186 | AGTR2 | angiotensin II receptor type 2 |
|  | 3164 | NR4A1 | nuclear receptor subfamily 4 group A member 1 |
|  | 11132 | CAPN10 | calpain 10 |
|  | 7494 | XBP1 | X-box binding protein 1 |
|  | 9861 | PSMD6 | proteasome 26S subunit, non-ATPase 6 |
|  | 162 | AP1B1 | adaptor related protein complex 1 subunit beta 1 |
|  | 4125 | MAN2B1 | mannosidase alpha class 2B member 1 |
|  | 1910 | EDNRB | endothelin receptor type B |
|  | 4162 | MCAM | melanoma cell adhesion molecule |
|  | 9235 | IL32 | interleukin 32 |
|  | 80781 | COL18A1 | collagen type XVIII alpha 1 chain |
|  | 6239 | RREB1 | ras responsive element binding protein 1 |
|  | 1356 | CP | ceruloplasmin |
|  | 6548 | SLC9A1 | solute carrier family 9 member A1 |
|  | 1435 | CSF1 | colony stimulating factor 1 |
|  | 5627 | PROS1 | protein S |
|  | 9133 | CCNB2 | cyclin B2 |
|  | 4254 | KITLG | KIT ligand |
|  | 5915 | RARB | retinoic acid receptor beta |
|  | 3676 | ITGA4 | integrin subunit alpha 4 |
|  | 51548 | SIRT6 | sirtuin 6 |
|  | 64805 | P2RY12 | purinergic receptor P2Y12 |
|  | 1012 | CDH13 | cadherin 13 |
|  | 2624 | GATA2 | GATA binding protein 2 |
|  | 1634 | DCN | decorin |
|  | 5445 | PON2 | paraoxonase 2 |
|  | 760 | CA2 | carbonic anhydrase 2 |
|  | 51129 | ANGPTL4 | angiopoietin like 4 |
|  | 727 | C5 | complement C5 |
|  | 59 | ACTA2 | actin alpha 2, smooth muscle |
|  | 2169 | FABP2 | fatty acid binding protein 2 |
|  | 10365 | KLF2 | Kruppel like factor 2 |
|  | 80279 | CDK5RAP3 | CDK5 regulatory subunit associated protein 3 |
|  | 6283 | S100A12 | S100 calcium binding protein A12 |
|  | 7391 | USF1 | upstream transcription factor 1 |
|  | 407007 | MIR222 | microRNA 222 |
|  | 6404 | SELPLG | selectin P ligand |
|  | 1514 | CTSL | cathepsin L |
|  | 231 | AKR1B1 | aldo-keto reductase family 1 member B |
|  | 4853 | NOTCH2 | notch receptor 2 |
|  | 27035 | NOX1 | NADPH oxidase 1 |
|  | 57007 | ACKR3 | atypical chemokine receptor 3 |
|  | 3382 | ICA1 | islet cell autoantigen 1 |
|  | 1028 | CDKN1C | cyclin dependent kinase inhibitor 1C |
|  | 160851 | DGKH | diacylglycerol kinase eta |
|  | 6721 | SREBF2 | sterol regulatory element binding transcription factor 2 |
|  | 85440 | DOCK7 | dedicator of cytokinesis 7 |
|  | 2961 | GTF2E2 | general transcription factor IIE subunit 2 |
|  | 406910 | MIR125A | microRNA 125a |
|  | 1839 | HBEGF | heparin binding EGF like growth factor |
|  | 3156 | HMGCR | 3-hydroxy-3-methylglutaryl-CoA reductase |
|  | 51206 | GP6 | glycoprotein VI platelet |
|  | 6402 | SELL | selectin L |
|  | 4040 | LRP6 | LDL receptor related protein 6 |
|  | 5052 | PRDX1 | peroxiredoxin 1 |
|  | 6736 | SRY | sex determining region Y |
|  | 2660 | MSTN | myostatin |
|  | 406942 | MIR150 | microRNA 150 |
|  | 2023 | ENO1 | enolase 1 |
|  | 706 | TSPO | translocator protein |
|  | 1513 | CTSK | cathepsin K |
|  | 2081 | ERN1 | endoplasmic reticulum to nucleus signaling 1 |
|  | 55000 | TUG1 | taurine up-regulated 1 |
|  | 4192 | MDK | midkine |
|  | 30009 | TBX21 | T-box transcription factor 21 |
|  | 1002 | CDH4 | cadherin 4 |
|  | 3567 | IL5 | interleukin 5 |
|  | 1118 | CHIT1 | chitinase 1 |
|  | 60674 | GAS5 | growth arrest specific 5 |
|  | 58191 | CXCL16 | C-X-C motif chemokine ligand 16 |
|  | 5598 | MAPK7 | mitogen-activated protein kinase 7 |
|  | 4547 | MTTP | microsomal triglyceride transfer protein |
|  | 1767 | DNAH5 | dynein axonemal heavy chain 5 |
|  | 2621 | GAS6 | growth arrest specific 6 |
|  | 8660 | IRS2 | insulin receptor substrate 2 |
|  | 942 | CD86 | CD86 molecule |
|  | 652995 | UCA1 | urothelial cancer associated 1 |
|  | 6898 | TAT | tyrosine aminotransferase |
|  | 406950 | MIR16-1 | microRNA 16-1 |
|  | 1909 | EDNRA | endothelin receptor type A |
|  | 54778 | RNF111 | ring finger protein 111 |
|  | 7292 | TNFSF4 | TNF superfamily member 4 |
|  | 10911 | UTS2 | urotensin 2 |
|  | 574501 | MIR499A | microRNA 499a |
|  | 407029 | MIR30A | microRNA 30a |
|  | 9032 | TM4SF5 | transmembrane 4 L six family member 5 |
|  | 22848 | AAK1 | AP2 associated kinase 1 |
|  | 4864 | NPC1 | NPC intracellular cholesterol transporter 1 |
|  | 8882 | ZPR1 | ZPR1 zinc finger |
|  | 1520 | CTSS | cathepsin S |
|  | 7414 | VCL | vinculin |
|  | 56477 | CCL28 | C-C motif chemokine ligand 28 |
|  | 10628 | TXNIP | thioredoxin interacting protein |
|  | 6509 | SLC1A4 | solute carrier family 1 member 4 |
|  | 51094 | ADIPOR1 | adiponectin receptor 1 |
|  | 336 | APOA2 | apolipoprotein A2 |
|  | 4321 | MMP12 | matrix metallopeptidase 12 |
|  | 9423 | NTN1 | netrin 1 |
|  | 9475 | ROCK2 | Rho associated coiled-coil containing protein kinase 2 |
|  | 57142 | RTN4 | reticulon 4 |
|  | 1311 | COMP | cartilage oligomeric matrix protein |
|  | 4256 | MGP | matrix Gla protein |
|  | 406989 | MIR206 | microRNA 206 |
|  | 4086 | SMAD1 | SMAD family member 1 |
|  | 6461 | SHB | SH2 domain containing adaptor protein B |
|  | 7498 | XDH | xanthine dehydrogenase |
|  | 2739 | GLO1 | glyoxalase I |
|  | 54751 | FBLIM1 | filamin binding LIM protein 1 |
|  | 2918 | GRM8 | glutamate metabotropic receptor 8 |
|  | 7296 | TXNRD1 | thioredoxin reductase 1 |
|  | 11082 | ESM1 | endothelial cell specific molecule 1 |
|  | 80332 | ADAM33 | ADAM metallopeptidase domain 33 |
|  | 145264 | SERPINA12 | serpin family A member 12 |
|  | 4929 | NR4A2 | nuclear receptor subfamily 4 group A member 2 |
|  | 2170 | FABP3 | fatty acid binding protein 3 |
|  | 653361 | NCF1 | neutrophil cytosolic factor 1 |
|  | 6037 | RNASE3 | ribonuclease A family member 3 |
|  | 241 | ALOX5AP | arachidonate 5-lipoxygenase activating protein |
|  | 2358 | FPR2 | formyl peptide receptor 2 |
|  | 3783 | KCNN4 | potassium calcium-activated channel subfamily N member 4 |
|  | 5008 | OSM | oncostatin M |
|  | 9966 | TNFSF15 | TNF superfamily member 15 |
|  | 821 | CANX | calnexin |
|  | 54898 | ELOVL2 | ELOVL fatty acid elongase 2 |
|  | 54796 | BNC2 | basonuclin 2 |
|  | 3578 | IL9 | interleukin 9 |
|  | 4773 | NFATC2 | nuclear factor of activated T cells 2 |
|  | 7980 | TFPI2 | tissue factor pathway inhibitor 2 |
|  | 2875 | GPT | glutamic--pyruvic transaminase |
|  | 7096 | TLR1 | toll like receptor 1 |
|  | 8398 | PLA2G6 | phospholipase A2 group VI |
|  | 54 | ACP5 | acid phosphatase 5, tartrate resistant |
|  | 2701 | GJA4 | gap junction protein alpha 4 |
|  | 6356 | CCL11 | C-C motif chemokine ligand 11 |
|  | 1235 | CCR6 | C-C motif chemokine receptor 6 |
|  | 6319 | SCD | stearoyl-CoA desaturase |
|  | 407024 | MIR29B1 | microRNA 29b-1 |
|  | 7350 | UCP1 | uncoupling protein 1 |
|  | 2693 | GHSR | growth hormone secretagogue receptor |
|  | 2328 | FMO3 | flavin containing dimethylaniline monoxygenase 3 |
|  | 3762 | KCNJ5 | potassium inwardly rectifying channel subfamily J member 5 |
|  | 9536 | PTGES | prostaglandin E synthase |
|  | 720 | C4A | complement C4A (Rodgers blood group) |
|  | 8218 | CLTCL1 | clathrin heavy chain like 1 |
|  | 407015 | MIR26A1 | microRNA 26a-1 |
|  | 8563 | THOC5 | THO complex 5 |
|  | 406934 | MIR142 | microRNA 142 |
|  | 9734 | HDAC9 | histone deacetylase 9 |
|  | 5291 | PIK3CB | phosphatidylinositol-4,5-bisphosphate 3-kinase catalytic subunit beta |
|  | 406903 | MIR10B | microRNA 10b |
|  | 9229 | DLGAP1 | DLG associated protein 1 |
|  | 29851 | ICOS | inducible T cell costimulator |
|  | 215 | ABCD1 | ATP binding cassette subfamily D member 1 |
|  | 3339 | HSPG2 | heparan sulfate proteoglycan 2 |
|  | 10461 | MERTK | MER proto-oncogene, tyrosine kinase |
|  | 3488 | IGFBP5 | insulin like growth factor binding protein 5 |
|  | 3959 | LGALS3BP | galectin 3 binding protein |
|  | 2202 | EFEMP1 | EGF containing fibulin extracellular matrix protein 1 |
|  | 50848 | F11R | F11 receptor |
|  | 406932 | MIR140 | microRNA 140 |
|  | 57761 | TRIB3 | tribbles pseudokinase 3 |
|  | 406979 | MIR19A | microRNA 19a |
|  | 9290 | GPR55 | G protein-coupled receptor 55 |
|  | 79602 | ADIPOR2 | adiponectin receptor 2 |
|  | 4208 | MEF2C | myocyte enhancer factor 2C |
|  | 26585 | GREM1 | gremlin 1, DAN family BMP antagonist |
|  | 6095 | RORA | RAR related orphan receptor A |
|  | 6361 | CCL17 | C-C motif chemokine ligand 17 |
|  | 10152 | ABI2 | abl interactor 2 |
|  | 57674 | RNF213 | ring finger protein 213 |
|  | 3250 | HPR | haptoglobin-related protein |
|  | 3604 | TNFRSF9 | TNF receptor superfamily member 9 |
|  | 9507 | ADAMTS4 | ADAM metallopeptidase with thrombospondin type 1 motif 4 |
|  | 1289 | COL5A1 | collagen type V alpha 1 chain |
|  | 2053 | EPHX2 | epoxide hydrolase 2 |
|  | 239 | ALOX12 | arachidonate 12-lipoxygenase, 12S type |
|  | 9388 | LIPG | lipase G, endothelial type |
|  | 406936 | MIR144 | microRNA 144 |
|  | 4643 | MYO1E | myosin IE |
|  | 407048 | MIR92A1 | microRNA 92a-1 |
|  | 55908 | ANGPTL8 | angiopoietin like 8 |
|  | 54567 | DLL4 | delta like canonical Notch ligand 4 |
|  | 127255 | LRRIQ3 | leucine rich repeats and IQ motif containing 3 |
|  | 7094 | TLN1 | talin 1 |
|  | 9641 | IKBKE | inhibitor of nuclear factor kappa B kinase subunit epsilon |
|  | 5730 | PTGDS | prostaglandin D2 synthase |
|  | 406941 | MIR149 | microRNA 149 |
|  | 5029 | P2RY2 | purinergic receptor P2Y2 |
|  | 57016 | AKR1B10 | aldo-keto reductase family 1 member B10 |
|  | 2357 | FPR1 | formyl peptide receptor 1 |
|  | 114815 | SORCS1 | sortilin related VPS10 domain containing receptor 1 |
|  | 9785 | DHX38 | DEAH-box helicase 38 |
|  | 2171 | FABP5 | fatty acid binding protein 5 |
|  | 4638 | MYLK | myosin light chain kinase |
|  | 9508 | ADAMTS3 | ADAM metallopeptidase with thrombospondin type 1 motif 3 |
|  | 1717 | DHCR7 | 7-dehydrocholesterol reductase |
|  | 406919 | MIR130A | microRNA 130a |
|  | 1374 | CPT1A | carnitine palmitoyltransferase 1A |
|  | 23358 | USP24 | ubiquitin specific peptidase 24 |
|  | 6414 | SELENOP | selenoprotein P |
|  | 27340 | UTP20 | UTP20 small subunit processome component |
|  | 5184 | PEPD | peptidase D |
|  | 58157 | NGB | neuroglobin |
|  | 633 | BGN | biglycan |
|  | 64241 | ABCG8 | ATP binding cassette subfamily G member 8 |
|  | 64581 | CLEC7A | C-type lectin domain containing 7A |
|  | 146664 | MGAT5B | alpha-1,6-mannosylglycoprotein 6-beta-N-acetylglucosaminyltransferase B |
|  | 6957 | TRB | T cell receptor beta locus |
|  | 79660 | PPP1R3B | protein phosphatase 1 regulatory subunit 3B |
|  | 3995 | FADS3 | fatty acid desaturase 3 |
|  | 3183 | HNRNPC | heterogeneous nuclear ribonucleoprotein C |
|  | 6367 | CCL22 | C-C motif chemokine ligand 22 |
|  | 8618 | CADPS | calcium dependent secretion activator |
|  | 1593 | CYP27A1 | cytochrome P450 family 27 subfamily A member 1 |
|  | 7942 | TFEB | transcription factor EB |
|  | 4481 | MSR1 | macrophage scavenger receptor 1 |
|  | 7058 | THBS2 | thrombospondin 2 |
|  | 10014 | HDAC5 | histone deacetylase 5 |
|  | 50863 | NTM | neurotrimin |
|  | 574456 | MIR497 | microRNA 497 |
|  | 9510 | ADAMTS1 | ADAM metallopeptidase with thrombospondin type 1 motif 1 |
|  | 4240 | MFGE8 | milk fat globule EGF and factor V/VIII domain containing |
|  | 1889 | ECE1 | endothelin converting enzyme 1 |
|  | 57591 | MRTFA | myocardin related transcription factor A |
|  | 123 | PLIN2 | perilipin 2 |
|  | 4758 | NEU1 | neuraminidase 1 |
|  | 3026 | HABP2 | hyaluronan binding protein 2 |
|  | 79400 | NOX5 | NADPH oxidase 5 |
|  | 51330 | TNFRSF12A | TNF receptor superfamily member 12A |
|  | 10333 | TLR6 | toll like receptor 6 |
|  | 8013 | NR4A3 | nuclear receptor subfamily 4 group A member 3 |
|  | 7252 | TSHB | thyroid stimulating hormone subunit beta |
|  | 8764 | TNFRSF14 | TNF receptor superfamily member 14 |
|  | 344 | APOC2 | apolipoprotein C2 |
|  | 152926 | PPM1K | protein phosphatase, Mg2+/Mn2+ dependent 1K |
|  | 407019 | MIR27B | microRNA 27b |
|  | 8754 | ADAM9 | ADAM metallopeptidase domain 9 |
|  | 8767 | RIPK2 | receptor interacting serine/threonine kinase 2 |
|  | 56910 | STARD7 | StAR related lipid transfer domain containing 7 |
|  | 10725 | NFAT5 | nuclear factor of activated T cells 5 |
|  | 325 | APCS | amyloid P component, serum |
|  | 8858 | PROZ | protein Z, vitamin K dependent plasma glycoprotein |
|  | 1075 | CTSC | cathepsin C |
|  | 9722 | NOS1AP | nitric oxide synthase 1 adaptor protein |
|  | 84634 | KISS1R | KISS1 receptor |
|  | 7177 | TPSAB1 | tryptase alpha/beta 1 |
|  | 187 | APLNR | apelin receptor |
|  | 6480 | ST6GAL1 | ST6 beta-galactoside alpha-2,6-sialyltransferase 1 |
|  | 29881 | NPC1L1 | NPC1 like intracellular cholesterol transporter 1 |
|  | 5687 | PSMA6 | proteasome 20S subunit alpha 6 |
|  | 5284 | PIGR | polymeric immunoglobulin receptor |
|  | 6646 | SOAT1 | sterol O-acyltransferase 1 |
|  | 623 | BDKRB1 | bradykinin receptor B1 |
|  | 8639 | AOC3 | amine oxidase copper containing 3 |
|  | 2729 | GCLC | glutamate-cysteine ligase catalytic subunit |
|  | 2114 | ETS2 | ETS proto-oncogene 2, transcription factor |
|  | 406943 | MIR152 | microRNA 152 |
|  | 1056 | CEL | carboxyl ester lipase |
|  | 643 | CXCR5 | C-X-C motif chemokine receptor 5 |
|  | 2658 | GDF2 | growth differentiation factor 2 |
|  | 7436 | VLDLR | very low density lipoprotein receptor |
|  | 4319 | MMP10 | matrix metallopeptidase 10 |
|  | 4056 | LTC4S | leukotriene C4 synthase |
|  | 9446 | GSTO1 | glutathione S-transferase omega 1 |
|  | 23303 | KIF13B | kinesin family member 13B |
|  | 3693 | ITGB5 | integrin subunit beta 5 |
|  | 221692 | PHACTR1 | phosphatase and actin regulator 1 |
|  | 4659 | PPP1R12A | protein phosphatase 1 regulatory subunit 12A |
|  | 728655 | HULC | hepatocellular carcinoma up-regulated long non-coding RNA |
|  | 7130 | TNFAIP6 | TNF alpha induced protein 6 |
|  | 10800 | CYSLTR1 | cysteinyl leukotriene receptor 1 |
|  | 11096 | ADAMTS5 | ADAM metallopeptidase with thrombospondin type 1 motif 5 |
|  | 407011 | MIR23B | microRNA 23b |
|  | 10663 | CXCR6 | C-X-C motif chemokine receptor 6 |
|  | 1373 | CPS1 | carbamoyl-phosphate synthase 1 |
|  | 407039 | MIR33A | microRNA 33a |
|  | 406884 | MIRLET7B | microRNA let-7b |
|  | 4060 | LUM | lumican |
|  | 23576 | DDAH1 | dimethylarginine dimethylaminohydrolase 1 |
|  | 3037 | HAS2 | hyaluronan synthase 2 |
|  | 7169 | TPM2 | tropomyosin 2 |
|  | 4048 | LTA4H | leukotriene A4 hydrolase |
|  | 23452 | ANGPTL2 | angiopoietin like 2 |
|  | 6955 | TRA | T cell receptor alpha locus |
|  | 4567 | MT-TL1 | mitochondrially encoded tRNA leucine 1 (UUA/G) |
|  | 94103 | ORMDL3 | ORMDL sphingolipid biosynthesis regulator 3 |
|  | 950 | SCARB2 | scavenger receptor class B member 2 |
|  | 970 | CD70 | CD70 molecule |
|  | 9027 | NAT8 | N-acetyltransferase 8 (putative) |
|  | 406994 | MIR212 | microRNA 212 |
|  | 100316868 | HOTTIP | HOXA distal transcript antisense RNA |
|  | 969 | CD69 | CD69 molecule |
|  | 4090 | SMAD5 | SMAD family member 5 |
|  | 345611 | IRGM | immunity related GTPase M |
|  | 7349 | UCN | urocortin |
|  | 23266 | ADGRL2 | adhesion G protein-coupled receptor L2 |
|  | 83667 | SESN2 | sestrin 2 |
|  | 64240 | ABCG5 | ATP binding cassette subfamily G member 5 |
|  | 10019 | SH2B3 | SH2B adaptor protein 3 |
|  | 4055 | LTBR | lymphotoxin beta receptor |
|  | 5740 | PTGIS | prostaglandin I2 synthase |
|  | 411 | ARSB | arylsulfatase B |
|  | 147495 | APCDD1 | APC down-regulated 1 |
|  | 8751 | ADAM15 | ADAM metallopeptidase domain 15 |
|  | 7041 | TGFB1I1 | transforming growth factor beta 1 induced transcript 1 |
|  | 3988 | LIPA | lipase A, lysosomal acid type |
|  | 1241 | LTB4R | leukotriene B4 receptor |
|  | 406961 | MIR185 | microRNA 185 |
|  | 23471 | TRAM1 | translocation associated membrane protein 1 |
|  | 407054 | MIR98 | microRNA 98 |
|  | 406902 | MIR10A | microRNA 10a |
|  | 968 | CD68 | CD68 molecule |
|  | 5446 | PON3 | paraoxonase 3 |
|  | 54957 | TXNL4B | thioredoxin like 4B |
|  | 3198 | HOXA1 | homeobox A1 |
|  | 384 | ARG2 | arginase 2 |
|  | 84159 | ARID5B | AT-rich interaction domain 5B |
|  | 10382 | TUBB4A | tubulin beta 4A class IVa |
|  | 406999 | MIR217 | microRNA 217 |
|  | 406915 | MIR128-1 | microRNA 128-1 |
|  | 55829 | SELENOS | selenoprotein S |
|  | 6916 | TBXAS1 | thromboxane A synthase 1 |
|  | 8614 | STC2 | stanniocalcin 2 |
|  | 9572 | NR1D1 | nuclear receptor subfamily 1 group D member 1 |
|  | 29904 | EEF2K | eukaryotic elongation factor 2 kinase |
|  | 286319 | TUSC1 | tumor suppressor candidate 1 |
|  | 23564 | DDAH2 | dimethylarginine dimethylaminohydrolase 2 |
|  | 10699 | CORIN | corin, serine peptidase |
|  | 22881 | ANKRD6 | ankyrin repeat domain 6 |
|  | 406955 | MIR181B1 | microRNA 181b-1 |
|  | 54386 | TERF2IP | TERF2 interacting protein |
|  | 1407 | CRY1 | cryptochrome circadian regulator 1 |
|  | 57491 | AHRR | aryl-hydrocarbon receptor repressor |
|  | 406980 | MIR19B1 | microRNA 19b-1 |
|  | 407055 | MIR99A | microRNA 99a |
|  | 3202 | HOXA5 | homeobox A5 |
|  | 80310 | PDGFD | platelet derived growth factor D |
|  | 442891 | MIR135B | microRNA 135b |
|  | 2294 | FOXF1 | forkhead box F1 |
|  | 1611 | DAP | death associated protein |
|  | 5641 | LGMN | legumain |
|  | 1363 | CPE | carboxypeptidase E |
|  | 50604 | IL20 | interleukin 20 |
|  | 5110 | PCMT1 | protein-L-isoaspartate (D-aspartate) O-methyltransferase |
|  | 101 | ADAM8 | ADAM metallopeptidase domain 8 |
|  | 406925 | MIR135A1 | microRNA 135a-1 |
|  | 91543 | RSAD2 | radical S-adenosyl methionine domain containing 2 |
|  | 406993 | MIR211 | microRNA 211 |
|  | 8718 | TNFRSF25 | TNF receptor superfamily member 25 |
|  | 10313 | RTN3 | reticulon 3 |
|  | 197259 | MLKL | mixed lineage kinase domain like pseudokinase |
|  | 406924 | MIR134 | microRNA 134 |
|  | 55553 | SOX6 | SRY-box transcription factor 6 |
|  | 1908 | EDN3 | endothelin 3 |
|  | 3053 | SERPIND1 | serpin family D member 1 |
|  | 10148 | EBI3 | Epstein-Barr virus induced 3 |
|  | 728215 | FAM155A | family with sequence similarity 155 member A |
|  | 140862 | ISM1 | isthmin 1 |
|  | 23022 | PALLD | palladin, cytoskeletal associated protein |
|  | 57705 | WDFY4 | WDFY family member 4 |
|  | 7301 | TYRO3 | TYRO3 protein tyrosine kinase |
|  | 771 | CA12 | carbonic anhydrase 12 |
|  | 3779 | KCNMB1 | potassium calcium-activated channel subfamily M regulatory beta subunit 1 |
|  | 9420 | CYP7B1 | cytochrome P450 family 7 subfamily B member 1 |
|  | 245973 | ATP6V1C2 | ATPase H+ transporting V1 subunit C2 |
|  | 407030 | MIR30B | microRNA 30b |
|  | 9456 | HOMER1 | homer scaffold protein 1 |
|  | 407031 | MIR30C1 | microRNA 30c-1 |
|  | 1240 | CMKLR1 | chemerin chemokine-like receptor 1 |
|  | 407034 | MIR30E | microRNA 30e |
|  | 3950 | LECT2 | leukocyte cell derived chemotaxin 2 |
|  | 1005 | CDH7 | cadherin 7 |
|  | 83700 | JAM3 | junctional adhesion molecule 3 |
|  | 27433 | TOR2A | torsin family 2 member A |
|  | 2849 | GPR26 | G protein-coupled receptor 26 |
|  | 440823 | MIAT | myocardial infarction associated transcript |
|  | 6489 | ST8SIA1 | ST8 alpha-N-acetyl-neuraminide alpha-2,8-sialyltransferase 1 |
|  | 114899 | C1QTNF3 | C1q and TNF related 3 |
|  | 8399 | PLA2G10 | phospholipase A2 group X |
|  | 10269 | ZMPSTE24 | zinc metallopeptidase STE24 |
|  | 746 | TMEM258 | transmembrane protein 258 |
|  | 27232 | GNMT | glycine N-methyltransferase |
|  | 56952 | PRTFDC1 | phosphoribosyl transferase domain containing 1 |
|  | 10488 | CREB3 | cAMP responsive element binding protein 3 |
|  | 56034 | PDGFC | platelet derived growth factor C |
|  | 10487 | CAP1 | cyclase associated actin cytoskeleton regulatory protein 1 |
|  | 51397 | COMMD10 | COMM domain containing 10 |
|  | 442892 | MIR148B | microRNA 148b |
|  | 54345 | SOX18 | SRY-box transcription factor 18 |
|  | 4648 | MYO7B | myosin VIIB |
|  | 8685 | MARCO | macrophage receptor with collagenous structure |
|  | 693120 | MIR33B | microRNA 33b |
|  | 3483 | IGFALS | insulin like growth factor binding protein acid labile subunit |
|  | 8482 | SEMA7A | semaphorin 7A (John Milton Hagen blood group) |
|  | 8490 | RGS5 | regulator of G protein signaling 5 |
|  | 10587 | TXNRD2 | thioredoxin reductase 2 |
|  | 5768 | QSOX1 | quiescin sulfhydryl oxidase 1 |
|  | 714 | C1QC | complement C1q C chain |
|  | 2837 | UTS2R | urotensin 2 receptor |
|  | 9563 | H6PD | hexose-6-phosphate dehydrogenase/glucose 1-dehydrogenase |
|  | 4969 | OGN | osteoglycin |
|  | 822 | CAPG | capping actin protein, gelsolin like |
|  | 57124 | CD248 | CD248 molecule |
|  | 574443 | MIR490 | microRNA 490 |
|  | 7837 | PXDN | peroxidasin |
|  | 10252 | SPRY1 | sprouty RTK signaling antagonist 1 |
|  | 406890 | MIRLET7G | microRNA let-7g |
|  | 2180 | ACSL1 | acyl-CoA synthetase long chain family member 1 |
|  | 338442 | HCAR2 | hydroxycarboxylic acid receptor 2 |
|  | 3957 | LGALS2 | galectin 2 |
|  | 4150 | MAZ | MYC associated zinc finger protein |
|  | 53630 | BCO1 | beta-carotene oxygenase 1 |
|  | 5050 | PAFAH1B3 | platelet activating factor acetylhydrolase 1b catalytic subunit 3 |
|  | 81796 | SLCO5A1 | solute carrier organic anion transporter family member 5A1 |
|  | 2941 | GSTA4 | glutathione S-transferase alpha 4 |
|  | 406895 | MIR103A1 | microRNA 103a-1 |
|  | 2350 | FOLR2 | folate receptor beta |
|  | 29953 | TRHDE | thyrotropin releasing hormone degrading enzyme |
|  | 56603 | CYP26B1 | cytochrome P450 family 26 subfamily B member 1 |
|  | 8315 | BRAP | BRCA1 associated protein |
|  | 10797 | MTHFD2 | methylenetetrahydrofolate dehydrogenase (NADP+ dependent) 2, methenyltetrahydrofolate cyclohydrolase |
|  | 345456 | PFN3 | profilin 3 |
|  | 6289 | SAA2 | serum amyloid A2 |
|  | 60482 | SLC5A7 | solute carrier family 5 member 7 |
|  | 6368 | CCL23 | C-C motif chemokine ligand 23 |
|  | 64902 | AGXT2 | alanine--glyoxylate aminotransferase 2 |
|  | 57823 | SLAMF7 | SLAM family member 7 |
|  | 11126 | CD160 | CD160 molecule |
|  | 5049 | PAFAH1B2 | platelet activating factor acetylhydrolase 1b catalytic subunit 2 |
|  | 57509 | MTUS1 | microtubule associated scaffold protein 1 |
|  | 375449 | MAST4 | microtubule associated serine/threonine kinase family member 4 |
|  | 9723 | SEMA3E | semaphorin 3E |
|  | 54809 | SAMD9 | sterile alpha motif domain containing 9 |
|  | 2928 | GSC2 | goosecoid homeobox 2 |
|  | 407049 | MIR92A2 | microRNA 92a-2 |
|  | 219699 | UNC5B | unc-5 netrin receptor B |
|  | 4519 | MT-CYB | mitochondrially encoded cytochrome b |
|  | 29116 | MYLIP | myosin regulatory light chain interacting protein |
|  | 55008 | HERC6 | HECT and RLD domain containing E3 ubiquitin protein ligase family member 6 |
|  | 283377 | SPRYD4 | SPRY domain containing 4 |
|  | 51177 | PLEKHO1 | pleckstrin homology domain containing O1 |
|  | 84666 | RETNLB | resistin like beta |
|  | 50487 | PLA2G3 | phospholipase A2 group III |
|  | 8993 | PGLYRP1 | peptidoglycan recognition protein 1 |
|  | 26253 | CLEC4E | C-type lectin domain family 4 member E |
|  | 7760 | ZNF213 | zinc finger protein 213 |
|  | 339761 | CYP27C1 | cytochrome P450 family 27 subfamily C member 1 |
|  | 407032 | MIR30C2 | microRNA 30c-2 |
|  | 114897 | C1QTNF1 | C1q and TNF related 1 |
|  | 8613 | PLPP3 | phospholipid phosphatase 3 |
|  | 4332 | MNDA | myeloid cell nuclear differentiation antigen |
|  | 4815 | NINJ2 | ninjurin 2 |
|  | 23263 | MCF2L | MCF.2 cell line derived transforming sequence like |
|  | 375056 | MIA3 | MIA SH3 domain ER export factor 3 |
|  | 11081 | KERA | keratocan |
|  | 338872 | C1QTNF9 | C1q and TNF related 9 |
|  | 93978 | CLEC6A | C-type lectin domain containing 6A |
|  | 9732 | DOCK4 | dedicator of cytokinesis 4 |
|  | 145957 | NRG4 | neuregulin 4 |
|  | 54499 | TMCO1 | transmembrane and coiled-coil domains 1 |
|  | 114882 | OSBPL8 | oxysterol binding protein like 8 |
|  | 5988 | RFPL1 | ret finger protein like 1 |
|  | 9620 | CELSR1 | cadherin EGF LAG seven-pass G-type receptor 1 |
|  | 474354 | LRRC18 | leucine rich repeat containing 18 |
|  | 407020 | MIR28 | microRNA 28 |
|  | 389643 | NUGGC | nuclear GTPase, germinal center associated |
|  | 114876 | OSBPL1A | oxysterol binding protein like 1A |
|  | 23762 | OSBP2 | oxysterol binding protein 2 |
|  | 83660 | TLN2 | talin 2 |
|  | 57787 | MARK4 | microtubule affinity regulating kinase 4 |
|  | 9760 | TOX | thymocyte selection associated high mobility group box |
|  | 728441 | GGT2 | gamma-glutamyltransferase 2 |
|  | 9455 | HOMER2 | homer scaffold protein 2 |
|  | 100874054 | FALEC | focally amplified long non-coding RNA in epithelial cancer |
|  | 10174 | SORBS3 | sorbin and SH3 domain containing 3 |
|  | 114004396 | LNCRNA-ATB | long noncoding RNA activated by TGF-beta |
|  | 166929 | SGMS2 | sphingomyelin synthase 2 |
|  | 374864 | CCDC178 | coiled-coil domain containing 178 |
|  | 91624 | NEXN | nexilin F-actin binding protein |
|  | 5136 | PDE1A | phosphodiesterase 1A |
|  | 29966 | STRN3 | striatin 3 |
|  | 3899 | AFF3 | AF4/FMR2 family member 3 |
|  | 56670 | SUCNR1 | succinate receptor 1 |
|  | 3083 | HGFAC | HGF activator |
|  | 126969 | SLC44A3 | solute carrier family 44 member 3 |
|  | 57552 | NCEH1 | neutral cholesterol ester hydrolase 1 |
|  | 79651 | RHBDF2 | rhomboid 5 homolog 2 |
|  | 574030 | MIR362 | microRNA 362 |
|  | 84830 | ADTRP | androgen dependent TFPI regulating protein |
|  | 1465 | CSRP1 | cysteine and glycine rich protein 1 |
|  | 128240 | NAXE | NAD(P)HX epimerase |
|  | 3171 | FOXA3 | forkhead box A3 |
|  | 51274 | KLF3 | Kruppel like factor 3 |
|  | 83464 | APH1B | aph-1 homolog B, gamma-secretase subunit |
|  | 55454 | CSGALNACT2 | chondroitin sulfate N-acetylgalactosaminyltransferase 2 |
|  | 339479 | BRINP3 | BMP/retinoic acid inducible neural specific 3 |
|  | 26273 | FBXO3 | F-box protein 3 |
|  | 7180 | CRISP2 | cysteine rich secretory protein 2 |
|  | 90952 | ESAM | endothelial cell adhesion molecule |
|  | 51530 | ZC3HC1 | zinc finger C3HC-type containing 1 |
|  | 388581 | C1QTNF12 | C1q and TNF related 12 |
|  | 7753 | ZNF202 | zinc finger protein 202 |
|  | 100270680 | CASC11 | cancer susceptibility 11 |
|  | 406926 | MIR135A2 | microRNA 135a-2 |
|  | 91012 | CERS5 | ceramide synthase 5 |
|  | 83953 | FCAMR | Fc fragment of IgA and IgM receptor |
|  | 116835 | HSPA12B | heat shock protein family A (Hsp70) member 12B |
|  | 375759 | C9orf50 | chromosome 9 open reading frame 50 |
|  | 157273 | LOC157273 | uncharacterized LOC157273 |
|  | 641516 | KC6 | keratoconus gene 6 |
|  | 57608 | JCAD | junctional cadherin 5 associated |
|  | 79987 | SVEP1 | sushi, von Willebrand factor type A, EGF and pentraxin domain containing 1 |
|  | 100302234 | MIR664A | microRNA 664a |
|  | 10100 | TSPAN2 | tetraspanin 2 |
|  | 284207 | METRNL | meteorin like, glial cell differentiation regulator |
|  | 406981 | MIR19B2 | microRNA 19b-2 |
|  | 23589 | CARHSP1 | calcium regulated heat stable protein 1 |
|  | 84888 | SPPL2A | signal peptide peptidase like 2A |
|  | 400360 | C15orf54 | chromosome 15 putative open reading frame 54 |
|  | 125206 | SLC5A10 | solute carrier family 5 member 10 |
|  | 56928 | SPPL2B | signal peptide peptidase like 2B |
|  | 388323 | GLTPD2 | glycolipid transfer protein domain containing 2 |
|  | 503497 | MS4A13 | membrane spanning 4-domains A13 |
|  | 360160 | CYCSP8 | CYCS pseudogene 8 |
|  | 100507053 | LOC100507053 | uncharacterized LOC100507053 |
|  | 129656 | CRTC1P1 | CRTC1 pseudogene 1 |
|  | 81695 | OR2B7P | olfactory receptor family 2 subfamily B member 7 pseudogene |
|  | 100507458 | ZNF213-AS1 | ZNF213 antisense RNA 1 (head to head) |
|  | 353329 | PSIP1P1 | PC4 and SFRS1 interacting protein 1 pseudogene 1 |
|  | 101926892 | LOC101926892 | uncharacterized LOC101926892 |
|  | 100130177 | LOC100130177 | uracil DNA glycosylase pseudogene |
|  | 729217 | LOC729217 | EBP like pseudogene |
|  | 149844 | LOC149844 | synaptotagmin binding cytoplasmic RNA interacting protein pseudogene |
|  | 9079 | LDB2 | LIM domain binding 2 |
|  | 102800311 | TP53COR1 | tumor protein p53 pathway corepressor 1 |
|  | 63036 | CELA2A | chymotrypsin like elastase 2A |
|  | 81786 | TRIM7 | tripartite motif containing 7 |
|  | 55911 | APOBR | apolipoprotein B receptor |
|  | 257313 | UTS2B | urotensin 2B |
|  | 11019 | LIAS | lipoic acid synthetase |
|  | 80350 | LPAL2 | lipoprotein(a) like 2, pseudogene |
|  | 222698 | NKAPL | NFKB activating protein like |
|  | 26577 | PCOLCE2 | procollagen C-endopeptidase enhancer 2 |
|  | 100133311 | HOXA-AS3 | HOXA cluster antisense RNA 3 |
|  | 65056 | GPBP1 | GC-rich promoter binding protein 1 |
|  | 401494 | HACD4 | 3-hydroxyacyl-CoA dehydratase 4 |
|  | 100422833 | MIR3188 | microRNA 3188 |
|  | 339789 | LINC00299 | long intergenic non-protein coding RNA 299 |
|  | 374987 | NEXN-AS1 | NEXN antisense RNA 1 |
|  | 100302209 | MIR1185-2 | microRNA 1185-2 |
|  | 100302157 | MIR1185-1 | microRNA 1185-1 |
|  | 157627 | MIR124-1HG | MIR124-1 host gene |
|  | 100144632 | HPBP | phosphate binding apolipoprotein |
|  | 104326055 | APOA1-AS | APOA1 antisense RNA |
|  | 108868751 | GSC-DT | GSC divergent transcript |
