# Supplementary material for: Network analysis of atherosclerotic genes elucidates druggable targets
Source: BMC Med Genomics. 2022 Mar 3;15:42. doi: 10.1186/s12920-022-01195-y (PMC8893053; doi:10.1186/s12920-022-01195-y)
Supplement: Supplementary file 8 — Additional file 8. Clusters generated after MCODE analysis. [file 12920_2022_1195_MOESM8_ESM.docx]

**Figure 1: Clusters generated after MCODE analysis. In each cluster the genes are represented by the coloured nodes and the interactions among them are represented by the edges.**


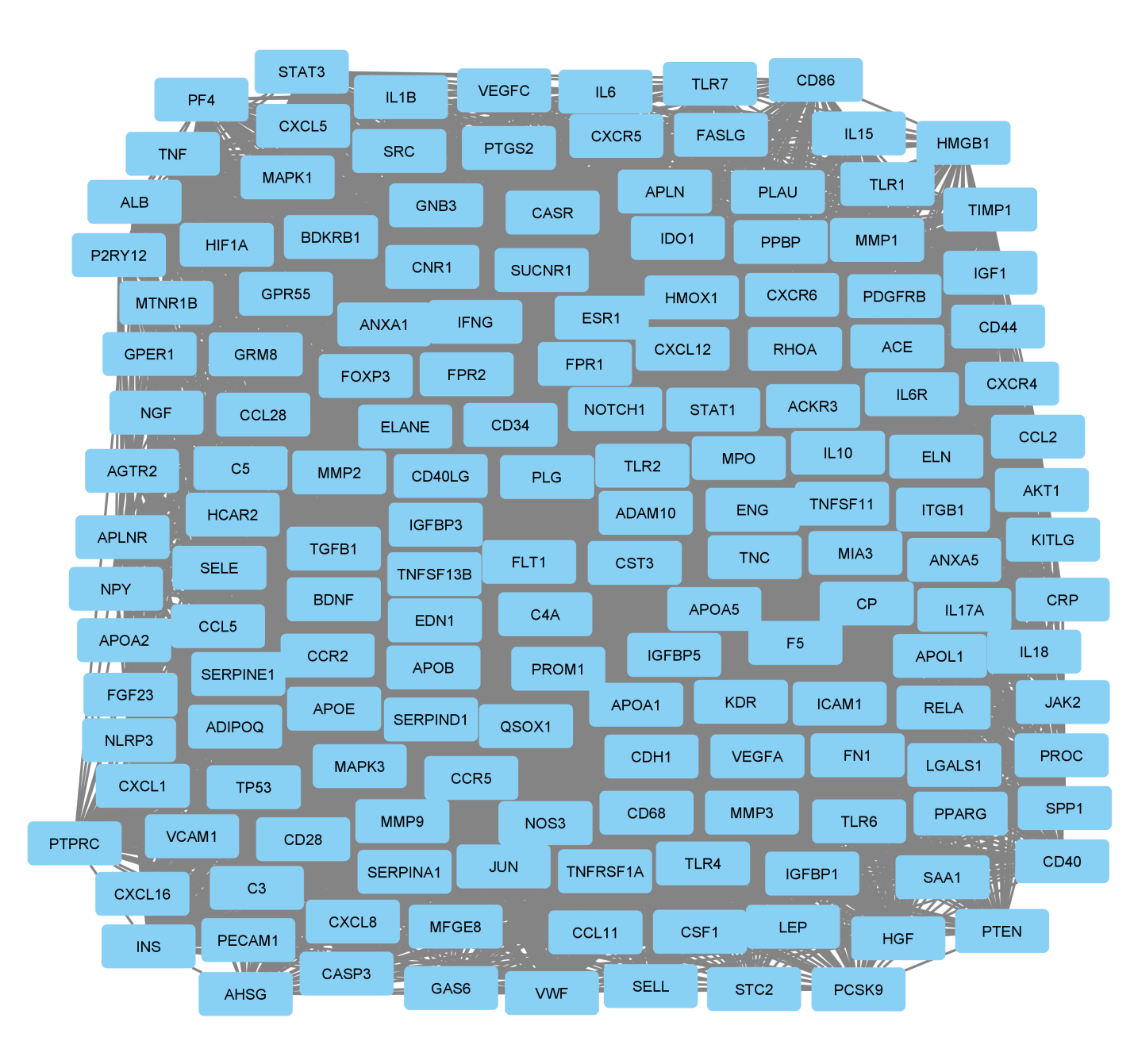


**Cluster1**


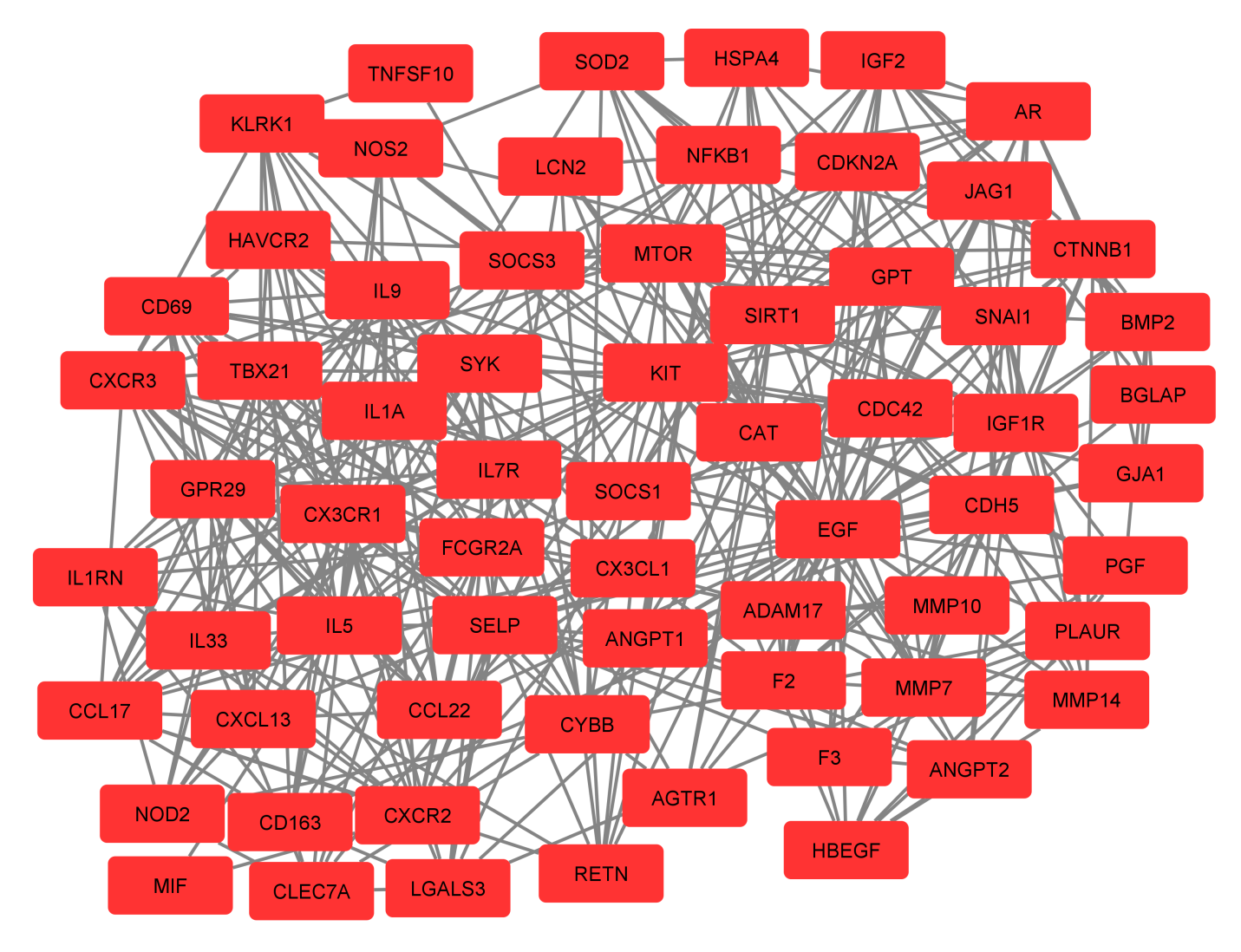


**Cluster 2**


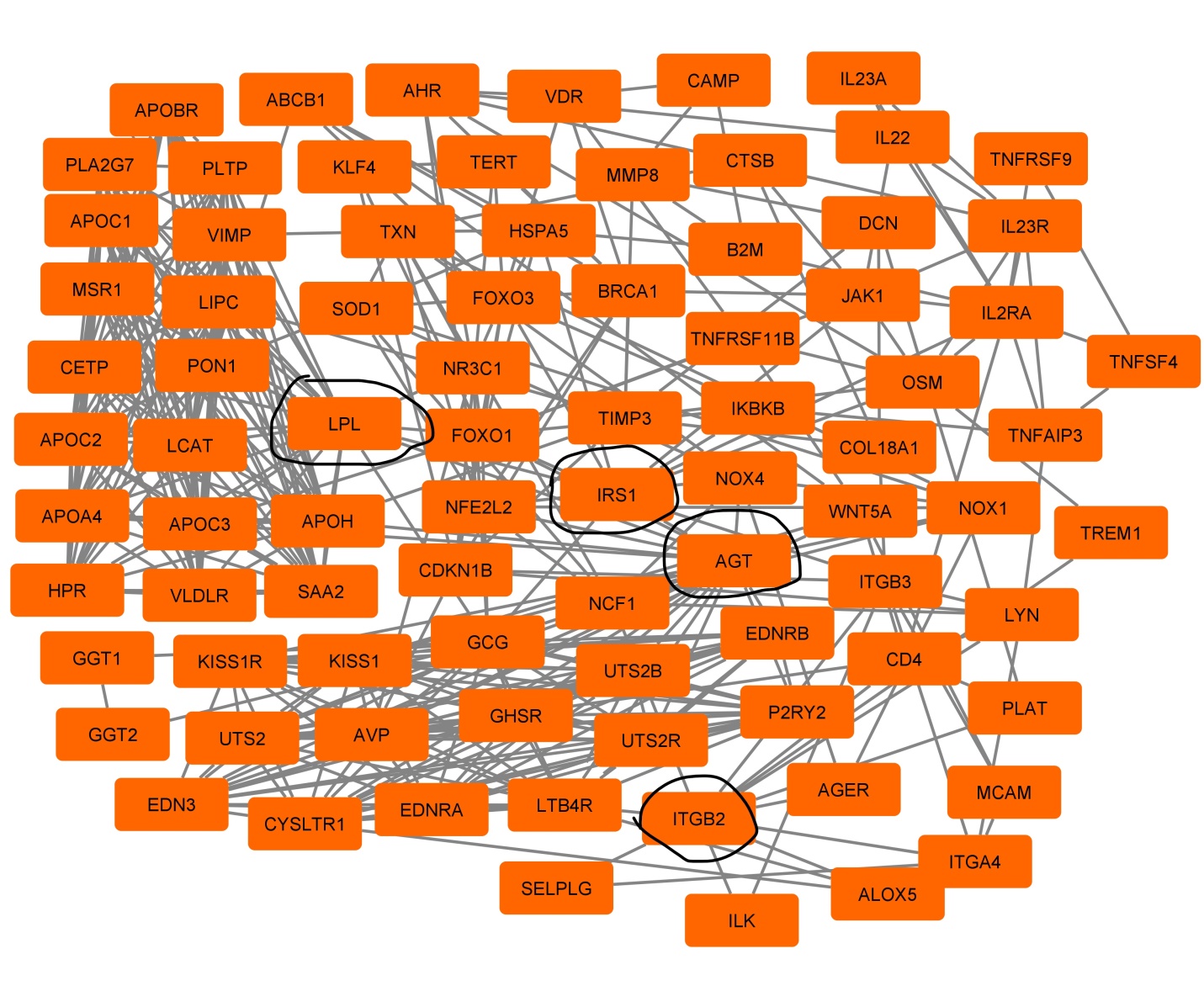


**Cluster 3**


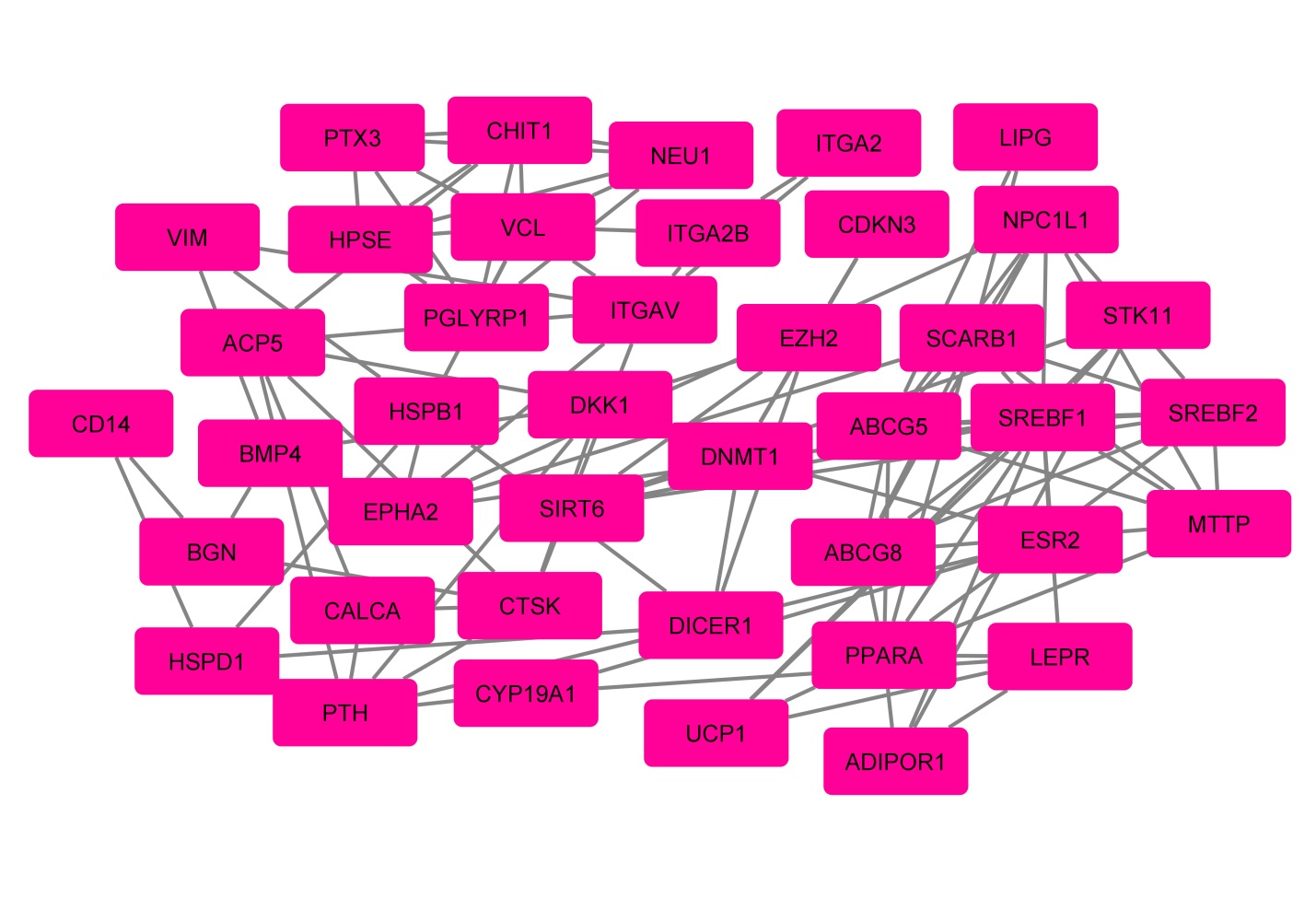


**Cluster 4**


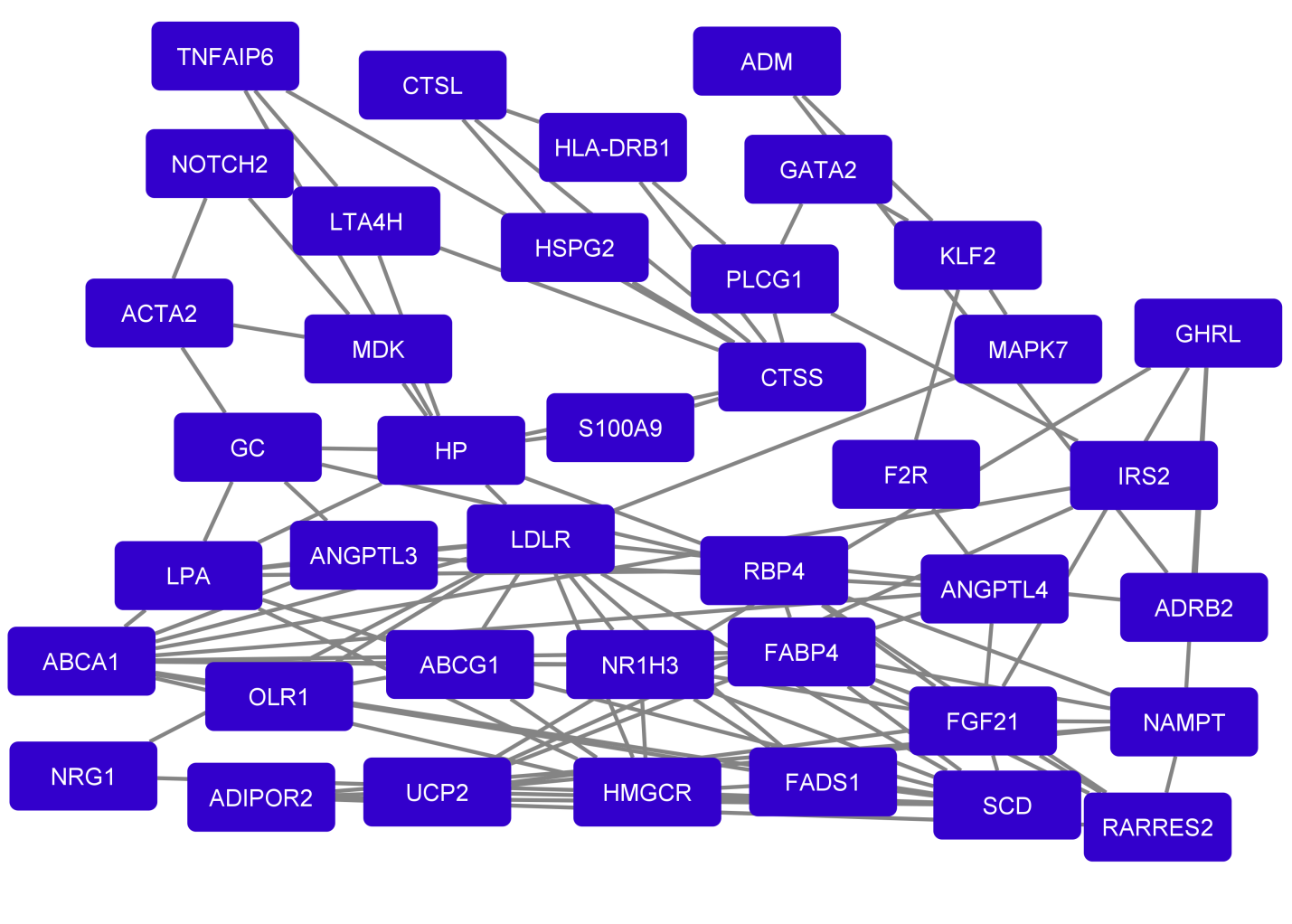


**Cluster 5**


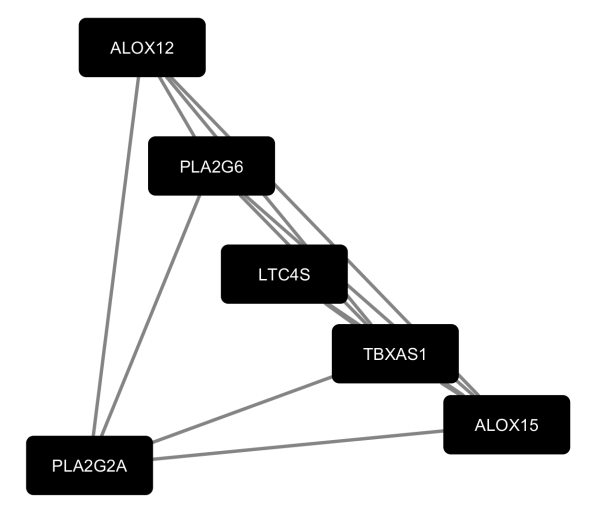

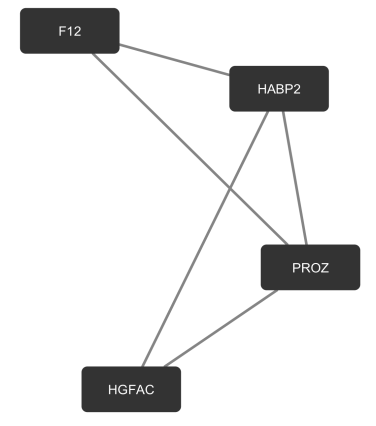


**Cluster 7**

**Cluster 6**


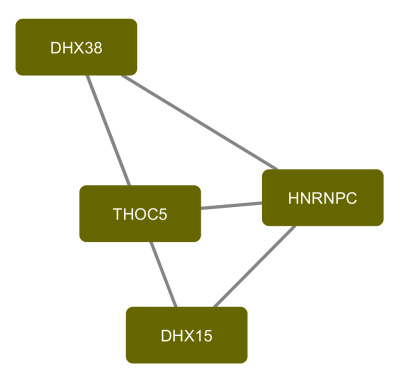

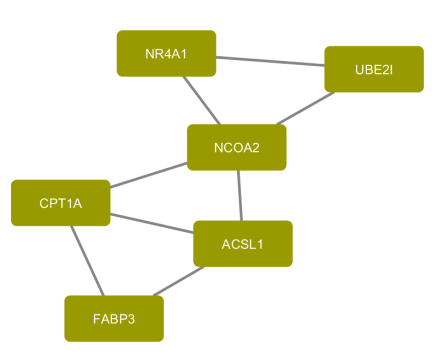


**Cluster 9**

**Cluster 8**


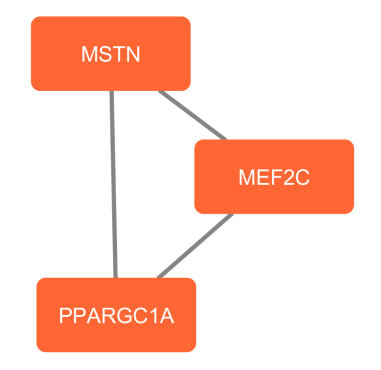

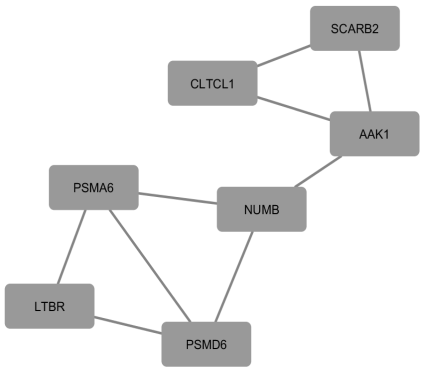


**Cluster 10**

**Cluster 11**


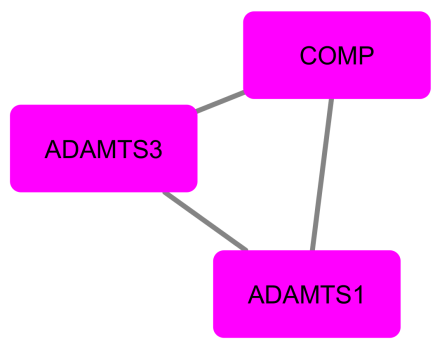

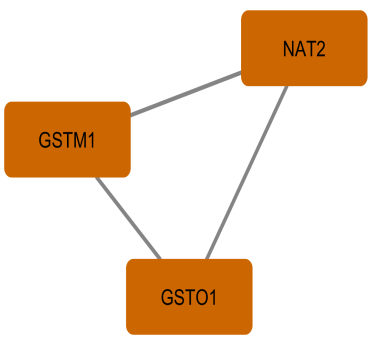


**Cluster 13**

**Cluster 12**


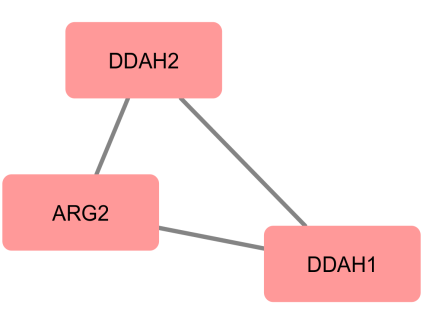

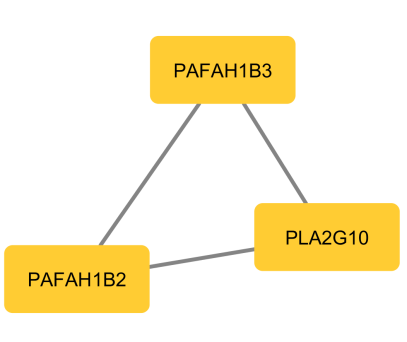


**Cluster 15**

**Cluster 14**


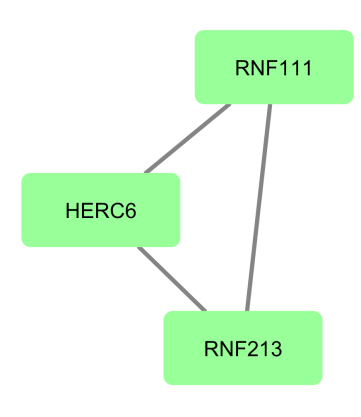

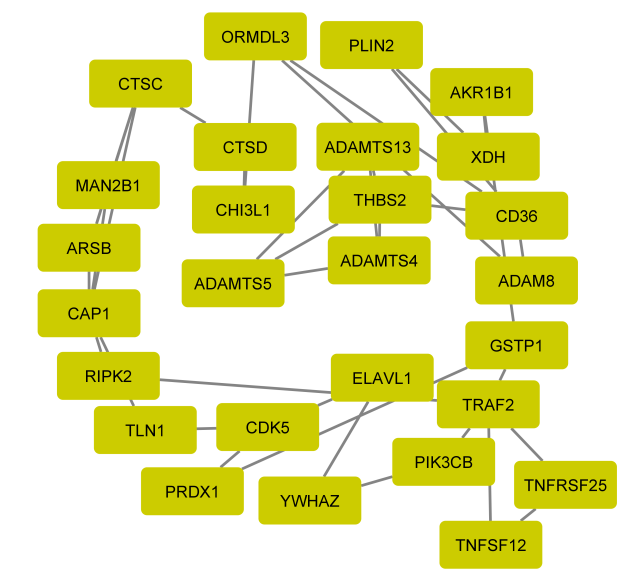


**Cluster 17**

**Cluster 16**


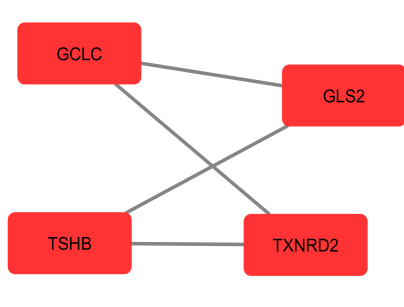


**Cluster 18**
